# Electrospray Ionization Mass Spectrometry: A Technique to Access the Information beyond the Molecular Weight of the Analyte

**DOI:** 10.1155/2012/282574

**Published:** 2011-12-15

**Authors:** Shibdas Banerjee, Shyamalava Mazumdar

**Affiliations:** Department of Chemical Sciences, Tata Institute of Fundamental Research, Homi Bhabha Road, Colaba, Mumbai 400005, India

## Abstract

The Electrospray Ionization (ESI) is a soft ionization technique extensively used for production of gas phase ions (without fragmentation) of thermally labile large supramolecules. In the present review we have described the development of Electrospray Ionization mass spectrometry (ESI-MS) during the last 25 years in the study of various properties of different types of biological molecules. There have been extensive studies on the mechanism of formation of charged gaseous species by the ESI. Several groups have investigated the origin and implications of the multiple charge states of proteins observed in the ESI-mass spectra of the proteins. The charged analytes produced by ESI can be fragmented by activating them in the gas-phase, and thus tandem mass spectrometry has been developed, which provides very important insights on the structural properties of the molecule. The review will highlight recent developments and emerging directions in this fascinating area of research.

## 1. Introduction

The basic concepts of chemistry originated from the quantitative estimation (e.g., weighing) of the constituents in a chemical reaction during the period of Lavoisier more than 200 years ago. Since then the analytical measurement of masses of the samples continuously evolved through the gravimetric analysis to weighing a single atom/molecule using the modern instrument called mass spectrometer. In mass spectrometry, a particular state of matter called gaseous ionic state is studied by transferring the analytes from condensed phase to the gas phase followed by their ionization. The success of the study of gas-phase ion chemistry and its application has been driven by the continuous advancement of the mass spectrometric technique since the studies were performed by Thomson [1]. As a result the mass spectrometry has become one of the most sensitive analytical methods for the structural characterization of molecules. Before the development of ESI-MS, there were several ionization methods (electron ionization, chemical ionization, etc.), but none of them could be able to overcome the propensity of the analyte fragmentation. In the mid 1980s, it became indispensable to precisely measure the molecular mass of the biologically important supramolecules like proteins [2]. But the proteins are polar, nonvolatile, and thermally labile molecules. So the ionization of the proteins by conventional ionization methods could lead to structural destruction. Although a technique called fast atom bombardment (FAB) [3] was available that time for the ionization of the biological samples, this technique produces predominantly singly charged ions of the analyte and the method works best for smaller species of mass below about 1000 Da. However, the available mass analyzers could not measure the high *m/z* value of the singly charged high molecular weight proteins during those days. So the only way to analyze the protein mass was to digest the protein and then the analysis of the digest mixture by FAB-mass spectrometry.

All those problems were overcome in 1989 when Fenn introduced electrospray ionization, a soft ionization technique, to ionize intact chemical species (proteins) by multiple charging [4]. The ionization is soft in the sense that a very little residual energy is retained by the analyte, and generally no fragmentation occurs upon ionization. Not only that but also very weak noncovalent interactions are preserved in the gas phase [5]. Because of the multiple charging, the *m/z* values of the resulting ions become lower and fall in the mass ranges of all common mass analyzers. Thus ESI became very useful in the production of gas-phase ions from large biologically important macromolecules like proteins and nucleic acids, and their subsequent mass spectrometric analysis for structural characterization as well as their rapid identification on the basis of molecular mass, a very specific property of the analyte. Gradually a systematic analysis of proteins with the mass spectrometry as the central tool led to a discrete subject called “Proteomics,” one of the fastest growing research areas in the chemical sciences [6].

In 2002 Fenn, the inventor of ESI-MS shared the 4th Nobel Prize in mass spectrometry along with Koichi Tanaka (for the development of MALDI mass spectrometry, another soft ionization technique) and Kurt Wuthrich (for the work in NMR spectroscopy). “A few years ago the idea of making proteins or polymers “fly” by electrospray ionization (ESI) seemed as improbable as a flying elephant, but today it is a standard part of modern mass spectrometers” as stated by the Professor Fenn in his Nobel lecture [7]. Nowadays ESI-MS is not only being used as a balance to weigh protein molecules but also to gain a deeper understanding of the protein three-dimensional structures, noncovalent interaction, posttranslation modification, and amino acid sequence. Soft landing of the mass-selected multiply charged gaseous protein ions into liquids (after the mass spectrometric separation) was recently shown to retain the native structures and even the biological activities of some proteins [8, 9]. Although the development of ESI-MS has had a major impact in biology and proteomics, its application has extended to a broad range of analytes including polar organic [10], inorganic [11], and metal-organic complexes [12]. Recently ESI efficiency scale of the different organic molecules with different polarities has been developed [13, 14]. The best ESI response has been observed for the analytes with ionizable basic/acidic polar functional groups. High-performance liquid chromatography has been coupled with the ESI-MS for the molecular fractionation prior to mass-spectrometric analysis. Thus, HPLC/ESI-MS has become a very powerful technique capable of analyzing both small and large molecules of various polarities in a complex biological sample mixture.

Here we would briefly review the development of the ESI-MS technique in last two and half decades not only for the mass access but also for the detailed understanding of the structural properties of the analyte in the different aspects of chemistry and biology including the fundamentals of the ionization mechanisms.

## 2. The Historical Perspective

There is an interesting history behind the development of ESI-MS. Although the process electrospray was known more than hundred years ago [15], the actual thought process on ESI-MS was initiated by Professor Dole, a physical chemist at Northwestern University. Much of the Dole's research focused on the polymerization chemistry. In the 1960s he was trying to characterize the size as well as mass distribution of some synthetic polymers (originally polystyrene) by mass spectrometric technique. But that time the troubles he encountered were the lack of a suitable ionization system which can produce molecular ions (without decomposing their structures) in the gas phase from the highly nonvolatile synthetic polymers and also the unavailability of the suitable detector system which can probe the appearance of the large molecular ions with high *m/z* value. Accidently he discovered the existence of electrospray while visiting a car manufacturer, and he observed the car painting by a process called electrospray. Then he applied the electrospray process in the production of gas-phase polystyrene ions and their subsequent collection using a Faraday cage detector [16, 17]. Although their experiments showed that the electrospray is a very promising soft ionization (no fragmentation of the analyte) technique, no mass spectrometer was available that time to separate and detect the ions of polystyrene molecules.

**Figure 1:**
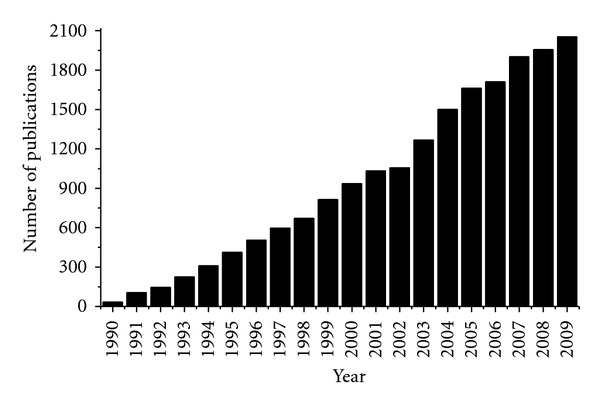
Yearly histogram of the papers dealing with the use of electrospray ionization after the Fenn's introduction of electrospray ionization mass spectrometry to ionize the biomolecules in 1989. The information was obtained by searching ISI Web of Knowledge on 26.06.2011 for the term “Electrospray ionization.”

However, Dole's report [16, 17] on electrospray got the attention of Professor Seymour Lipsky and Professor Csaba Horvath at Yale Medical School [2]. That time (1970s) Professor Lipsky was thinking about the alternate ways of ionizing biopolymers like proteins, and Professor Horvath was to work on the development of HPLC known as high-pressure liquid chromatography. They noticed that two of the Dole's reports referred the work of Fenn who was a professor in the Yale Engineering Department that time. Fenn was a specialist in the field of molecular beams and their production by nozzle-skimmer systems. Through those references Lipsky got in touch with Fenn. Fenn accepted the challenge of the production of biomolecular ions in the gas phase using his molecular beam apparatus even though he was approaching 65, typical retirement age [2]. Finally Fenn group's ground breaking discovery on the ionization and characterization of large biomolecules in the gas phase by electrospray ionization mass spectrometry [4] created a new dimension in the field of proteomics. After the Fenn's discovery of ESI-MS technique, the uses of the electrospray ionization continue to grow at an unprecedented rate (see Figure 1), and every day new applications are developed as the instrument continues to advance as fast as the need.

## 3. Basic Architecture of the ESI-Mass Spectrometer

**Figure 2:**
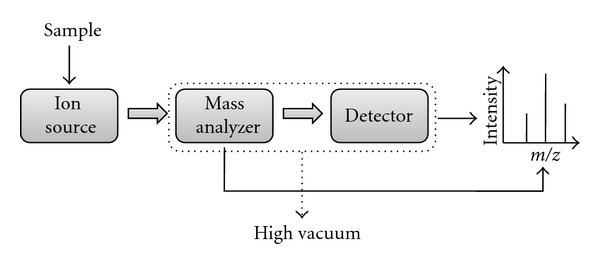
The basic components of the ESI-mass spectrometer.

Like other mass spectrometers, ESI-mass spectrometer is also composed of three basic components, for example, ion source, mass analyzer, and detector (see Figure 2). The intact molecular ions (not truly ions, see later) are produced in the ionization chamber where the ion source is kept, and then they are transferred in the mass analyzer region via several ion optics (electromagnetic elements like skimmer, focusing lens, multipole, etc.), which are basically kept to focus the ion stream to maintain a stable trajectory of the ions. The mass analyzer sorts and separates the ions according to their mass to charge ratio (*m/z* value). The separated ions are then passed to the detector systems to measure their concentration, and the results are displayed on a chart called a mass spectrum (see Figure 2). Since the ions in the gas phase are very reactive and often short lived, their formation and manipulation should be conducted in high vacuum. For this reason the ion optics, analyzer, and also the detectors are kept at very high vacuum (typically from 10^−3^ torr to 10^−6^ torr pressure). Mass spectrometers typically use either oil diffusion pumps or turbomolecular pumps to achieve the high vacuum required to operate the instrument. Generally the ion source is kept at atmospheric pressure, and a continuous pressure gradient and voltage gradient are used from source to the detector to help pump out the ions from source to the detector through the analyzer.

### 3.1. Ion Source

**Figure 3:**
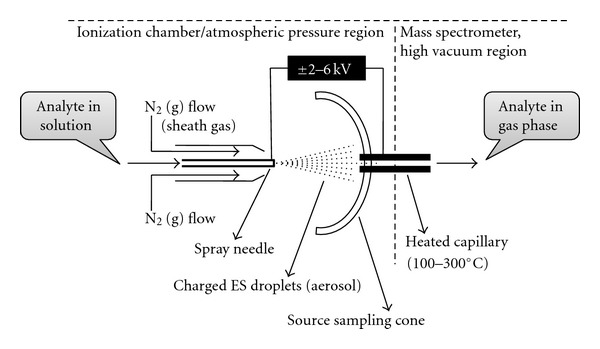
A schematic representation of the ESI-ion source.

A suitable ESI source for the mass-spectrometric analysis was designed by the Fenn group in the mid 1980s [4, 18–20]. Later on it was modified by different research groups to improve the system's robustness [21–25]. Generally a dilute (less than mM in polar volatile solvent) analyte solution is injected by a mechanical syringe pump through a hypodermic needle or stainless steel capillary (~0.2 mm o.d and ~0.1 mm i.d) at low flow rate (typically 1–20 *μ*L/min). A very high voltage (2–6 kV) is applied to the tip of the metal capillary relative to the surrounding source-sampling cone or heated capillary (typically located at 1–3 cm from the spray needle tip). This strong electric field causes the dispersion of the sample solution into an aerosol of highly charged electrospray (ES) droplets (see Figure 3). A coaxial sheath gas (dry N_2_) flow around the capillary results in better nebulization. This gas flow also helps to direct the spray emerging from the capillary tip towards the mass spectrometer. The charged droplets diminish in size by solvent evaporation, assisted by the flow of nitrogen (drying gas).

Finally the charged analytes are released from the droplets, some of which pass through a sampling cone or the orifice of a heated capillary (kept in the interface of atmospheric pressure and the high vacuum) into the analyser of the mass spectrometer, which is held under high vacuum. The heated capillary (typically 0.2 mm inner diameter, 60 mm in length and heated to 100–300°C) causes the complete desolvation of the ions passing through it. The use of drying gas and the heated capillary can influence the system's robustness and reduce the degree of cluster ion formation [24]. The transfer of analyte ions from solution to gas phase is not an energetic process, but rather the desolvation process effectively cools the gaseous ions. So the analyte ions with low internal energies are allowed to enter into the mass spectrometer from the electrospray probe, and the structure of the analytes generally remain intact (no fragmentation) when appropriate instrumental conditions (e.g., no activation of the ions in gas phase) are used. Nowadays a number of sprayer modifications like pneumatically assisted electrospray [26–28], ultrasonic nebulizer electrospray [29, 30], electrosonic spray [31], and nanoelectrospray [23, 32] have been developed to expand the range of ESI applications. Among them the most popular one is nanoelectrospray.

Nanospray ionization is a low flow rate (20–50 nL/min) version of electrospray ionization [32]. A very low sample concentration (nanomole/mL) and low volume are required for nanospraying. Such downscaling has been achieved by replacing the spray needle with borosilicate glass capillary of some microliters volume to which a fine tip (1–4 *μ*m inner diameter) is pulled with a micropipette puller. The spray voltage of 0.7–1.1 kV is normally applied via an electrically conducting coating (usually a sputtered gold film) on the outer surface of the spray capillary. When the high voltage is switched on, the analyte solution flow is solely driven by capillary forces refilling the aperture as droplets are leaving the tip. While conventional ESI generates initial charged droplets of 1-2 *μ*m in diameter, the nanospray produces the charged droplets of the less than 200 nm diameter; that is, their volume is about 100–1000 times smaller than the droplets produced by a conventional microemitter. The nano-ESI has an increased tolerance to high aqueous solvents and salt contamination [23, 32]. In this technique not only less analyte sample is consumed than with the standard electrospray ionization, but also a small volume of sample lasts for several minutes, thus enabling multiple experiments to be performed.

### 3.2. Mass Analyzer

The mass analyzer is the heart of the mass spectrometer. The mass analyzer can be compared with the prism. The component wavelengths of a light are separated by a prism, and then they are detected by an optical receptor. Similarly in the mass analyzer, the different types of ions (*m/z*) of an ion beam are separated, and then they are passed to the detector. There are many types of mass analyzers [33, 34], for example, magnetic (B)/electric (E) sector mass analyzer, linear quadrupole ion trap (LIT), three-dimensional quadrupole ion trap (QIT) [35], orbitrap, time-of-flight mass analyzer (TOF), and ion cyclotron resonance mass analyzer (ICR), all of these which use the static or dynamic magnetic/electric field, and all operate according to two fundamental laws of physics, for example, Lorentz force law and Newton's second law of motion. Proper selection of the mass analyzer depends on the resolution, mass range, scan rate, and detection limit required for an application. Although the detailed discussion of different types of mass analyzers is beyond the scope of this paper, interested readers can go through any standard mass spectrometry text book for this issue.

### 3.3. Detector

The simplest way to detect the ion is the use of Faraday cup, where the ion is allowed to be neutralized and the resulting current is measured. This Faraday cup is used when the ion flux is relatively large. But a more modern way to measure the low ion flux is the use of electron multiplier (just like photomultiplier tube to measure the photon flux) [36, 37]. The energetic ions are allowed to strike a metal or semiconductor plate (e.g., copper/beryllium alloy oxide) called a conversion dynode that emits secondary electrons (SE). This conversion dynode is held at very high voltage (order of kV). The secondary electrons emitted are accelerated and focused onto the second and subsequent dynodes (kept in positive potentials), which are set at potentials progressively closer to earth. At each dynode there is an increase in the number of electrons emitted (electron avalanche), such that at the end of the multiplier a gain of approximately 10^6^ is achieved. The output current is converted to a voltage signal, which finally can be translated to an intensity value (the ordinate axis of the mass spectrum) by means of an analog-to-digital (ADC) converter. There are several types of multipliers like discrete dynode electron multipliers [36, 37], channel electron multipliers (CEMs) [34], microchannel or multichannel plates (MCPs) [34], and so forth. Unlike other mass spectrometers, ions are not detected by hitting a detector such as an electron multiplier in FT-ICR-MS [38], but ions are detected by the measurement of the image current produced by ions cyclotroning in the presence of a magnetic field. A detector is selected according to its speed, dynamic range, gain, and geometry. Some detectors are sensitive enough to detect a single ion. Although there has been a revolution in the mass spectrometer development in the last twenty years by several researchers and companies, the question regarding the response of the detector haunts the researchers till now. How does the detector respond to the large multiply charged ions produced by ESI? No precise information is available regarding the fact whether the observed peak height corresponding to a multiply charged macroion reflects the number of incident ions, the number of charges they carry, the conformation of the ion, the energy of incidence, its velocity, or an unknown combination of these factors [39]. Though it is assumed that a peak height in a mass spectrum is directly proportional to the number of corresponding incident ions to the detector, this issue still remains suspicious as the detector response has not been characterized appropriately in those aspects mentioned above [39].

**Figure 4:**
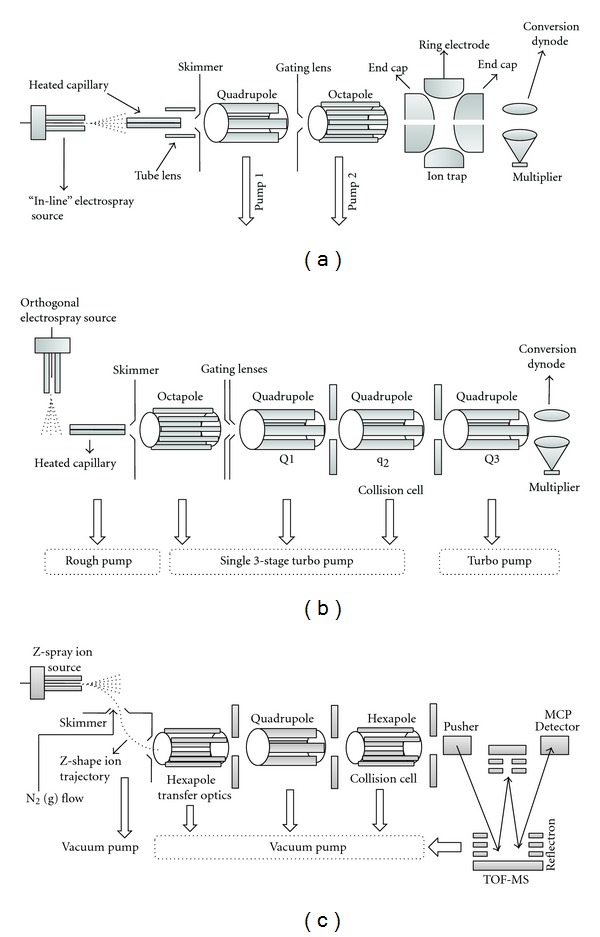
Schematic of the (a) thermo-Finnigan LCQ Deca mass spectrometer (on-axis spray), (b) Agilent 6410 Triple Quad LC/MS system (off-axis/orthogonal spray), and (c) Waters Micromass Q-TOF Ultima ESI-MS (z-spray).

Figure 4 shows some hybrid mass spectrometers (commercially available) which are constructed by combining different types of *m/z* separation devices or mass analyzers. Different types of detectors and spray (ion source) geometries are also noticeable in those instruments (Figures 4(a)–4(c)). These instruments are specially designed for the tandem mass analysis (see later). The trajectory of the ions from ion source to the analyzer is linear (on-axis/line-of-sight) in conventional electrospray sources (Figure 4(a)). But, recently the spray geometries have been modified to orthogonal spray (Figure 4(b)) or z-spray (Figure 4(c)) where the ion trajectory from source to the analyzer is, respectively, orthogonal and z shaped. These off-axis spray geometries circumvent the problem of the clogging of heated capillaries and skimmers by neutral molecules, nonvolatile materials, and so forth.

## 4. The ESI-Mass Spectrum

**Figure 5:**
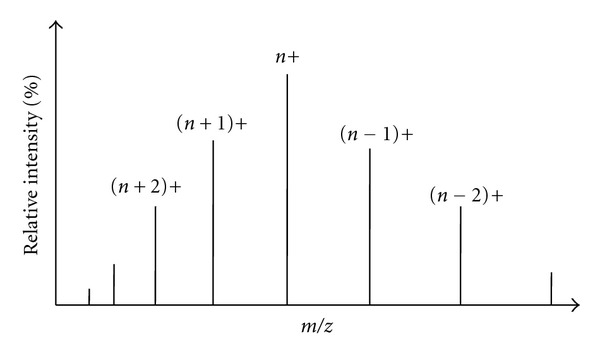
A typical cartoon representing the nature of ESI-mass spectrum in positive ion mode.

Generally the ions derived by ES process are multiply charged, and the analyte remains intact (no fragmentation) when appropriate instrumental conditions are used. In positive ion mode (when the spraying nozzle is kept at positive potential) the charging generally occurs via protonation (sometimes metalation also), but in negative ion mode (when the spraying nozzle is kept at negative potential) charging occurs via deprotonation of the analyte. The mechanism of charging has been discussed later. Since the charging of the analyte occurs by transfer of protons, the ionic species detected are not the true molecular ions (which are formed by the loss or gain of the electron), but they are more preferably protonated or deprotonated molecules [40]. When a pure analyte solution is electrosprayed all the peaks appearing in the corresponding ESI-mass spectrum represent the intact molecular species with variable charging as shown in Figure 5. The ordinate or vertical axis represents the relative abundances of multiply charged species of the same analyte, and the abscissa or horizontal axis represents the mass to charge ratio (*m/z*) of the multiply charged analyte. As usual the most intense or the tallest peak (peak of *n+* charge in Figure 5) with 100% relative abundance is called the “base peak.” The quantity *m/z* is dimensionless since it is the ration of two dimensionless quantities mass number (*m*) and the elementary charges (*z*). Sometimes the unit *Thomson *[Th] is applied to the *m/z* value to honor Thomson. Very often the symbol “u” (unified atomic mass) or Da (Dalton, in biological mass spectrometry field to honor J. Dalton) is also used as the molecular mass unit.

Now the question is how one can determine the molecular mass of the analyte with the help of the ion signals (different *m/z* values) related to the same molecule? The procedure of finding the charge and mass of the analyte through the ESI-mass spectrum is called “Mathematical Charge Deconvolution” [41–43]. The process is straightforward and based on the assumption that two adjacent peaks in an ESI-mass spectrum of a single analyte have charge state difference one as has been shown in Figure 5. If it is assumed that the molecular mass of the analyte is *M* and the observed *m/z* values for two neighboring charge states *n+* and (*n* − 1)+ are *m*_1_ and *m*_2_, respectively, then two mathematical equations can easily be drawn as (*M* + *n*)/*n* = *m*_1_ and [*M *+ (*n* − 1)]/(*n* − 1) = *m*_2_. Since there are only two unknown parameters *M* and *n*, a minimum of two equations are required to find out the values of *M* and *n*. So the above two equations can easily be employed to determine the value of *n* (charge state) and *M* (deconvoluted mass). One can think the observed multiple peaks as the multiple mass assessment of the same molecule. A more accurate mass of the analyte can be derived by averaging the calculated mass for each peak. The above algorithm works well for the pure analyte and when the analyte produces the successive charge states of the difference unity. But problems arise when the analyte produces metaliated as well as protonated species in gas phase or skips some charge state in between two observed charge states. Therefore, several refined procedures have been developed to cope with these requirements [44]. Nowadays all the modern ESI-MS instruments are equipped with elaborate software for charge deconvolution.

Proteins, the most popular analytes for ESI-MS study exhibit different charge state distribution profiles in their ESI-mass spectra. To quantify the charge state distribution of the analyte, the term “average charge state” (*Z*_av_) was introduced [45] and defined as ∑*z*_*i*_ · *I*_*i*_/∑*I*_*i*_, where *I*_*i*_ is the mass spectrometrically detected signal intensity of a given charge state (*z*_*i*_) carried by the *i*th ion. Latter on several correlations between this average charge state and the protein structures were found (discussed later).

## 5. The Mechanism of Electrospray Ionization

After the development of ESI-MS, many different assumptions and hypotheses were made in the early 1990s to interpret the multiple charging of the analyte by the ES process [46–49]. That time it was thought that the distribution of the charge states in the ESI-MS spectra actually reflects the degree of charging of the analyte (say proteins) in the neutral solution (as determined by solution phase equilibrium) [41, 50, 51]. But later it became evident that there is no correlation between solution charging and electrospray charging after the report of Kelly and coworkers [47]. Their report showed that in positive ion mode ESI produced protonated (positively charged) analyte (myoglobin) though the analyte is overall negatively charged (deprotonated) in basic aspirating solution (pH 10), and in negative ion mode it produced deprotonated (negatively charged) analyte (myoglobin) though the analyte is overall positively charged (protonated) in acidic aspirating solution (pH 3). These observations implied that the charging process in ESI might occur in an entirely different manner than that it was thought.

When an analyte is transferred from solution to the gas phase via ESI, the analyte solution undergoes three major processes. These are (a) production of the charged droplets from the high-voltage capillary tip where the analyte solution is injected; (b) repeated solvent evaporation (from the charged droplet) and droplet disintegration, resulting a very small charged droplet, which is able to produce the charged analyte; (c) finally a mechanism by which the gas-phase ion is formed. Since the electrospray process existed long before its application in the mass spectrometry and earlier it was used for the electrostatic dispersion of liquids and the creation of aerosol, the first two processes were mostly studied by the researchers in the aerosol science, and the processes are well understood nowadays [15, 52]. But the last process, that is, the mechanisms of the ion formation from vary small highly charged droplet is still under controversy, and the exact process happening at this stage is not unambiguously known. Over the last two decades, the issue has hotly been discussed and debated, and several hypotheses based on the theory and experimental evidences were invoked [22, 53, 54], and here we would discuss the most popular models of the gas-phase ion formation.

### 5.1. Production of Charged Droplets

**Figure 6:**
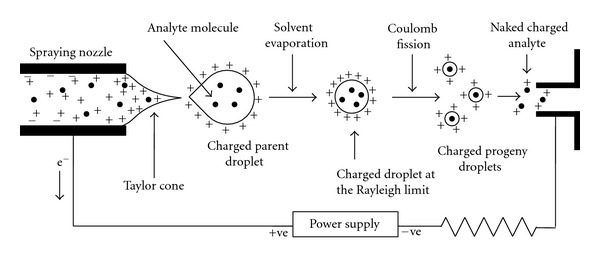
Schematic representation of the electrospray ionization process.

When the analyte solution is pumped through the high-voltage capillary (emitter), an electrochemical reaction of the solvent occurs which causes an electron flow to or from the metal capillary depending on its polarity [55]. In absence of any redox active analyte [56, 57], the oxidation of the solvent occurs in the positive ion mode and reduction of the solvent occurs in the negative ion mode as has been shown in Table 1 [58]. So the ES ion source could be “viewed as an electrolytic cell of a special kind” [56]. These redox reactions (Table 1) supply positive or negative ions in the solutions depending on the polarity of the emitter electrode. Generally polar solvents (e.g., water, methanol, acetonitrile, etc.), which easily undergo electrochemical reactions in the spraying nozzle, are used in the ESI-MS experiments [58]. The accumulated charges (positive or negative) would be repelled by the high-voltage capillary (of the same polarity), and then they would drift towards the liquid surface at the capillary outlet (see Figure 6). However, the accumulated like charges at the surface are destabilized, and finally the meniscus would be drawn out and deformed into a cone under the influenced of a very high electric field (typically ~10^6^ V/m) [22, 58]. This cone is called a Taylor cone [59] after the name of the scientist Taylor who first theoretically described the conditions under which a stable liquid cone can exist with the competing forces of an electric field and the surface tension of the liquid [59]. This Taylor cone is the zone of high turbulence. At high field strength, it immediately starts ejecting a fine jet of liquid from its apex towards the counter electrode (heated capillary) [60]. This charged jet easily breaks up into small droplets. The droplets produced at low flow rate (typically ~5 *μ*L/min) have a narrow distribution of sizes with a most abundant radius ~1.5 *μ*m [22]. Such an electrospray droplet was shown to have a charge of ~10^−14^ C, which corresponds to ~60,000 singly charged ions. All those charged droplets are driven away from each other by Coulombic repulsion and move along the direction of the electric fields (towards the heated capillary) (see Figure 6). Overall, under positive ion mode positively charged aerosol is formed and in negative ion mode negatively charged aerosol is formed. The positive charges are mostly contributed by protons, and negative charges are contributed by some negative ions (e.g., OH^−^) (see Table 1) [58].

### 5.2. Coulomb Explosion and Disintegration of the Charged Droplets

**Figure 7:**
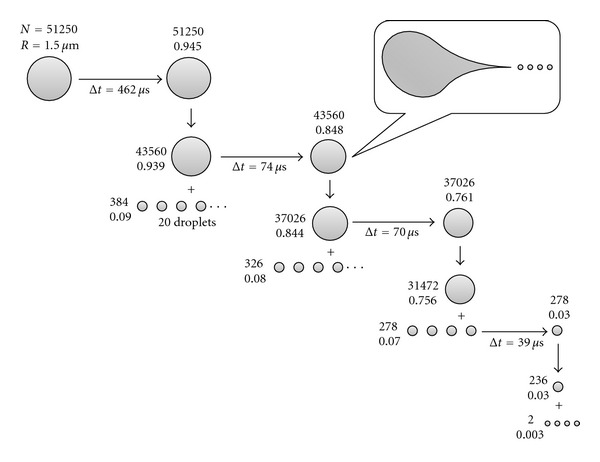
Time history of the charged methanol droplet produced by microelectrospray process. The droplet at the top left is a typical parent droplet created at the ES capillary tip. The successive solvent evaporation and Coulomb fission leads to the charged nanodroplets that are the precursors of the gas-phase analyte ions. The numbers beside the droplets give radius *R* (*μ*m) and number of elementary charges *N* on the ES droplet; Δ*t* corresponds to the time required for evaporative droplet shrinkage to size where fission occurs. Only the first three successive fissions of a parent droplet are shown. At the bottom right, the fission of the offspring droplet to produce the charged nanodroplets is shown. The inset shows a drawing of droplet jet fission based on actual flash microphotograph. (Adapted with permission from [22], Copyright 1993, American Chemical Society).

The charges (say protons in positive ion mode) in the droplets are distributed on its surface with equidistant spacing to minimize the potential energy [39]. There are two forces acting in opposite directions in the charged droplets. One is surface tension of the charged droplet, which tries to retain the spherical shape of the droplet, and the other is Coulomb force of repulsion between the like charges on the surface, which tries to break down the spherical shape of the charged droplet [22, 39, 54, 58]. The solvent evaporation occurs when the droplets traverse the space between spraying nozzle and the heated capillary (see Figure 6). As a result the size of the droplet decreases until it reaches the point (Rayleigh limit) [62] where the surface tension can no longer sustain the Coulomb force of repulsion, and at this point “Coulomb explosion” or “Coulomb fission” occurs; that is, the parent droplet disintegrates into much smaller offspring droplets. The emitted stream of the offspring/progeny droplets holds about 2% mass and 15% charge of the parent droplet [22]. So the offspring droplets are not only much smaller than their parent but also have much higher charge-to-mass ratio. The process of solvent evaporation and Coulomb fission occurs repeatedly to generate smaller and smaller progeny droplets and finally the charged nanodroplets from which the gas-phase charged analyte molecule is formed [22, 63].  Figure 7 describes the detail evolution of the initial droplets, formed from the Taylor cone, to the droplets that are the precursor of the gas-phase ions. Typically the flying microdroplets were observed to vibrate alternately from oblate to prolate shapes, and this elastic vibration causes the parent droplet to emit a tail of much smaller offspring droplets (droplet jet fission) [22, 53, 64] as has been shown in the inset of Figure 7. This disruption pattern is quite similar to the disruption at the Taylor cone. Therefore, the charge density on the droplet surface is not homogeneous, but significantly increased in the region of sharper curvature, and it has been shown the charged microdroplets fission somewhat before, at ~80% of the Rayleigh limit [22]. The concept of this charged droplet jet fission is based on theoretical as well as experimental evidence (flash microphotographs) [64–66]. The life time of an ES droplet largely dependent on several parameters like ion spray voltage, nature of the solvents, sheath gas flow rate, distance between the spraying nozzle and heated capillary and temperature of the heated capillary, and so forth, [22, 28] and that would in turn affect the charging of the analyte. However, the average life time of a charged droplet produced by the ES process is around one to a few milliseconds for typical interfaces that one would use in ESI-MS [22, 67].

### 5.3. Formation of Gas-Phase Analyte Ions from the Charged Droplets

 Two principle mechanisms have been proposed to account for the formation of gas-phase analyte ions from very small highly charged ES droplets, and those are *charge residue model* (CRM) [16, 68, 69] and *ion evaporation model* (IEM) [63].

#### 5.3.1. Charge Residue Model

As a result of a series of solvent evaporation and Coulomb fission, an extremely small charged droplet (*R≈* 1 nm) is formed which contains only one analyte molecule [16, 22]. Desolvation of this droplet causes its charges (on the surface) to land on the analyte molecules. Since the “residual” droplet charge at the last stage of the solvent evaporation in the ES process is retained by the analyte molecule in the gas phase, this mechanism is called charge residue mechanism/model. Originally this mechanism was hypothesized by Dole et al. [16] and a more detail consideration as well as support of this mechanism was given by Schmelzeisen-Redeker et al. [68].

A Study of Winger and coworkers showed that generally large macromolecules like proteins follow CRM [70]. They determined one interesting empirical correlation (see (1)) between the analyte molecular mass (*M*) and the observed average charge state (*Z*_av_) in the ESI-mss spectra of the corresponding analyte (starburst dendrimers) [71]. The “*a*” and “*b*” are constants and *b* = 0.53 gave the best result

(1) $$\;Z_{\text{av}}={aM}^{b}.$$

Later on Fernandez De La Mora [61] showed that the above equation (1) could be theoretically derived for the analytes like proteins that follow CRM. He assumed that after the final Coulomb fission event that produces the ultimate charged water droplet containing one neutral protein molecule would be a little bit larger in size than that of the protein. Then the final solvent evaporation would completely transfer the droplet charges to the protein. He also assumed that the density of the globular protein is the same as that of water (*ρ* = 1 g/c.c) [61, 72], and then he calculated the radius of the protein as

(2) $$\left(\frac{4}{3}\pi R^{3}\right)\rho N_{A}=M.$$

**Figure 8:**
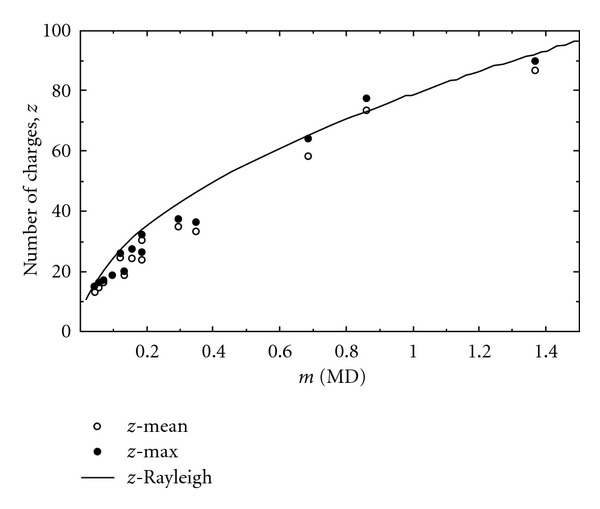
Plot of the average charge state (*z*) against molecular mass (*m*) for various proteins that follow CRM. [(•) Observed highest charge state; (*○*) observed lowest charge state; solid curve corresponds to the average charge state predicted by (4)]. The molecular weights of the proteins given on the *x*-axis are in units of 10^6^ Da. (Reprinted with permission from *Analytica Chimica Acta* [61], Copyright 2000, Elsevier).

The Rayleigh limit charging (which deals with the Rayleigh stability limit for coulomb fission of the charged droplet) of the water droplet having the same radius (*R*) as of the analyte protein can be given by the Rayleigh equation (3) [62], and then combining (2) and (3) he found the (4) [61] which is similar to (1) empirically obtained by Winger and coworkers. Notably that the exponent for *M* equals to 0.5 is very much close to the exponent 0.53 of the empirical equation (1)

(3) $$Z_{R}\cdot e=8\pi {\left(\varepsilon_{0}\gamma R^{3}\right)}^{1/2},$$

(4) $$Z_{R}=4{\left(\frac{\pi \gamma \varepsilon_{0}}{\rho e^{2}N_{A}}\right)}^{1/2}\times M^{1/2}=0.078M^{1/2},$$

where *e* is the elementary charge, *Z* is the charge number, *ε*_0_ is the permittivity of the surrounding medium, *N*_*A*_ is the Avogadro's number, *ρ* is the density of the water, *M* is the molecular weight of the protein, and *γ* is the surface tension of the solvent (water). A plot (solid line) shown in Figure 8 is based on the above theoretically derived equation (4), and that fits well with the empirical data (shown by circles) of several proteins investigated by Fernandez De La Mora. So the observed agreement between the charges on the proteins and the charges on water droplets of the same size, at the Rayleigh limit, is not a coincidence but a consequence of the multiply charged proteins being formed by the CRM.

#### 5.3.2. Ion Evaporation Model

After repeated solvent evaporation and Coulomb fission, the radii of the charged droplets decrease to a given size when the electric field due to the charges at the surface of the droplet is strong enough to cause direct emission of the solvated ions [22, 63, 73]. Typically when the droplet reaches the size *R* ≤ 10 nm, the ion emission dominates over Rayleigh fission. Thus unlike CRM, this mechanism does not require the production of very small droplets (*R* *≈* 1 nm) that contains only one analyte molecule. This mechanism is called ion evaporation mechanism/model as proposed by Iribarne and Thomson [22, 63, 73]. They proposed that the rate constant *k *_1_ for ion emission from the highly charged droplet surface can be given by the following equation (5) where *k*_*b*_ is the Boltzmann constant, *T* is the temperature of the droplet, *h* is the Planck constant, and Δ*G* ^†^ is the free energy of activation:

(5) $$k_{1}=\frac{k_{b}T}{h}\exp\left(-\frac{{\Delta G}^{†}}{RT}\right).$$

The activation energy is influenced by several parameters like (a) attraction between the escaping ion and the solvent by which the droplet is composed, (b) Coulomb repulsion of the escaping ion by the remaining charges on the droplet surface, and (c) ion desolvation energy. Although the above Iribarne and Thomson equation (5) can successfully interpret the ion evaporation process, it cannot predict the observed relative intensities of ions in the ESI mass spectra, which is mostly influenced by the structural and physicochemical nature of the analyte and the solvent used. However, IEM is experimentally well supported for small inorganic and organic ions [53, 61, 74, 75].

#### 5.3.3. Fenn's Model of Ion Formation: An Extension of IEM

**Figure 9:**
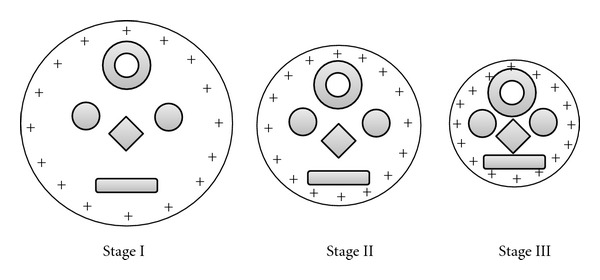
Hypothetical picture of a charge droplet containing five analytes of different shapes and sizes at three different stages of the solvent evaporation before Coulomb fission.

Unlike IEM, the Fenn's model [39, 69] states that the analytes remain neutral inside the neutral core of the charge droplet (charges lie on the surface). If the analyte is intrinsically charged (e.g., proteins), this charged neutrality could be maintained either by a nearby counter anion as its “shadow” (i.e., ion pair formation) or by a proton transfer process between analyte and the solvent. The overall neutral analyte would have a rapid Brownian diffusion motion inside the charged droplet. The successive solvent evaporation and Coulomb fission would continuously increase the surface charge density of the droplet. In positive ion mode these charges are basically protons. Protons are pretty small, and they can have a strong attractive interaction with the molecules of polar solvents. When a given part of the analyte encounters the proton-rich surface by Brownian thrashing (energy on the order of *k*_*b*_ *T*), the basic residues located at that portion of the analyte would be attached to the surface protons. Thus the protons would serve to anchor the analyte molecule nearer to the surface. Thermal activation (Brownian oscillation, internal vibration, and rotation, with amplitudes constrained by *k*_*b*_ *T*) may provide the energy for the analyte to move some distance outside the droplet. This separation between the protonated analyte residues and the charges on the droplet surface leads to repulsion, which facilitate the escape of a remaining portion of the molecule that carries other charge sites. With increase in the charged residues (outside the droplet surface) of the escaping analyte, the Coulomb repulsion increases and that facilitate the escape of the whole analyte with a varying degree of charging. The degree of charging varies not only for the size, shape, orientation, and fugacity or escaping tendency of the analyte but also for the continuously variable charge spacing and electric field at the droplet surface during the process of repeated solvent evaporation and Coulomb fission. Thus, the number of charges that are attached to the analyte molecule when it desorbs from the surface is the number of charges that it can span on the surface during its emission. The situation is illustrated in Figure 9, which shows three hypothetical snapshots of a charged droplet at different stages during its evaporation before undergoing Coulomb fission. The droplet contains five analytes of different shapes and sizes. The droplet size decreases with solvent evaporation and the distance between equally spaced positive charges decreases since the number of charges remains constant. Clearly, at the evaporation stage III, the desorption of the analytes from the droplet surface would lead more charging than that at the stage I. Again at each stage the charging will vary according to the conformation and size of the analyte. But in practice the ejection of the charged analyte would continuously lower the number of charges and number of analyte species in the host droplet from stage I to stage III. As a result, above and below some critical charge states, the relative ion abundance decreases and that is reflected by a bell-shaped distribution of the ion abundances (see Figure 5). Of course the scenario is much more complicated because of the involvement of the Coulomb fission since it also contributes the charge and analyte concentration in a droplet.

## 6. Factors That Influence the Charge State Distribution (CSD) of Proteins

**Figure 10:**
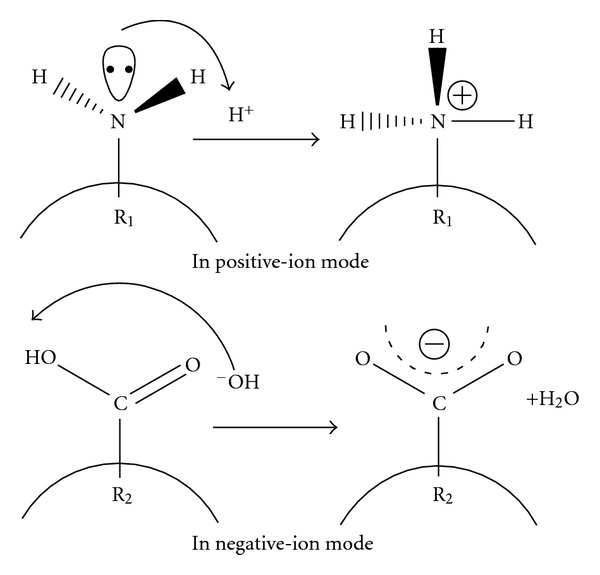
The proton transfer reaction responsible for protein charging in gas phase via ESI.

Multiple charging is an intrinsic feature of electrospray ionization of macromolecules like proteins [41–43]. Several factors affect the charge state distributions (CSDs) of proteins in vacuum generated by ESI [76]. Mostly the physical dimension of the protein molecules in solution are the major determinant of this CSD [76, 77]. As discussed earlier, in positive ion mode the basic sites in the proteins can accommodate protons and in the negative ion mode the acidic sites in the proteins can give up protons (see Figure 10) in the vanishing charged droplet and thus responsible for the gas-phase ion formation mostly via CRM [78]. Here we would discuss some critical factors known to significantly influence the protein CSD. 

### 6.1. Solution pH

The change of pH of the protein solution can change the protein conformation (tertiary structure), and the degree of the conformational change depends on the nature of the protein and the acidity/basicity of the solution. According to the CRM, it is the geometry or size of the protein that actually determines the average charge state (CS) of the electrosprayed protein in vacuum [76]. Relatively small number of charge states (low average CS) is produced when a natively folded protein is transferred to the gas phase upon ESI than that when an unfolded/denatured protein undergoes ESI. This is because the native proteins maintain a compact structure by its tightly folded polypeptide chain, and this compactness is lost upon denaturation. After denaturation, the solvent accessibility as well as the size of the protein increases, which in turn leads to increase the charge state distribution over a broad range (*m/z*) with the high average CS [45, 48, 76, 79].

**Figure 11:**
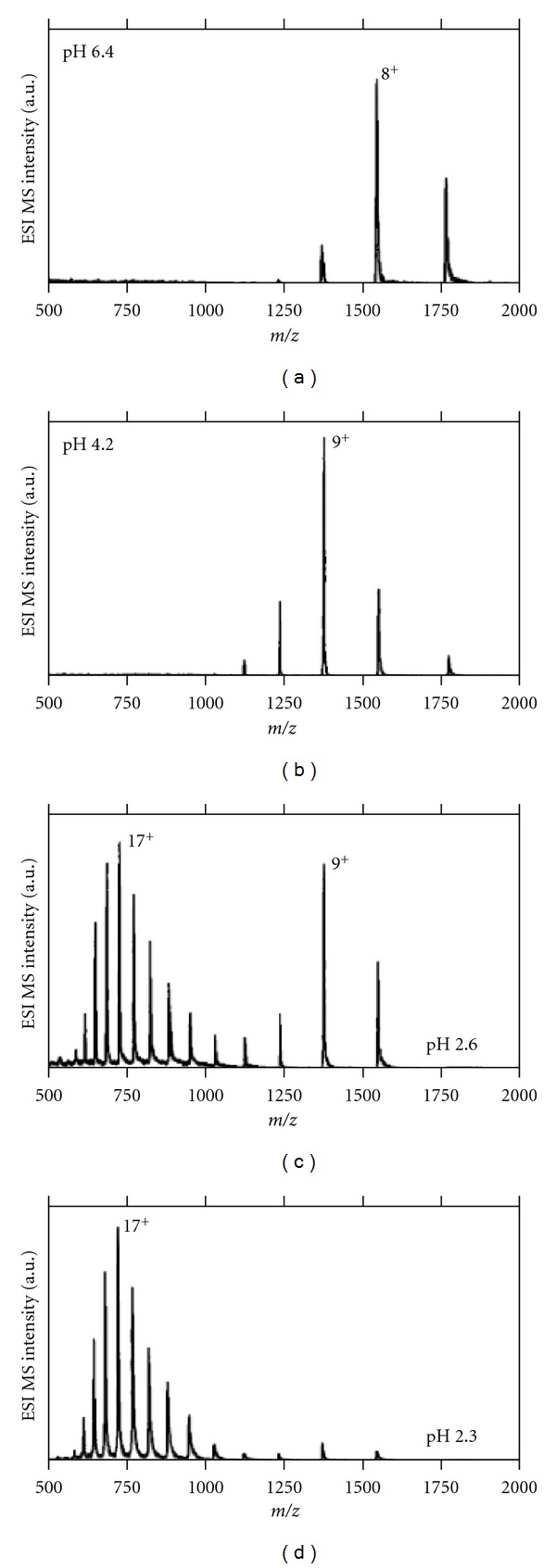
ESI-mass spectra of cyt c in water containing 3% methanol and 0.5 mM ammonium acetate at (a) pH 6.4, (b) pH 4.2, (c) pH 2.6, and (d) pH 2.3. The pH was adjusted by the addition of hydrochloric acid. (Reprinted with permission from *Biochemistry* [80], Copyright 1997, American Chemical Society).

Figure 11 shows a typical example of ESI-CSD of cytochrome c at different pH in positive ion mode [80]. At pH 6.4, where cyt c is known to adopt native, tightly folded conformation, the positive ion ESI-MS (Figure 11(a)) shows relatively narrow CSD with +8 as the most intense CS (base peak). But at pH 2.3, where a substantial unfolding of cyt c occurs, the positive ion ESI-MS (Figure 11(d)) shows a broad CSD with +17 as the most intense CS (base peak). Native and nonnative protein states often coexist at equilibrium under mildly denaturing conditions. In such conditions, the CSD becomes bimodal, reflecting the presence of both native and denatured states. At pH 2.6 cyt c exists in both native and unfolded states as is evidenced by the bimodal CSD in the ESI-mass spectrum (Figure 11(c)).

In certain cases proteins, for example, ubiquitin unfolds in both acidic (pH 2) and basic solution (pH 11.7) and when the protein was electrosprayed from both acidic and basic solution, the corresponding positive ion mass spectra showed characteristic broad CSD with maxima at +11 and +8, respectively, [81]. But the ESI-mass spectrum of ubiquitin in neutral solution (pH 7.2) [81] showed narrow CSD with most intense charge at +6.

The native environment of pepsin (in gastric juice) is extremely acidic, and at native pH (pH ≤ 2) it remains in folded active form. So when it is electrosprayed from the acidic solution (pH 1.6), it shows remarkably narrow CSD [82], and the CSD does not change until the solution pH is raised above 2.5, a threshold pH of pepsin deactivation [77]. The large scale unfolding of pepsin occurs in neutral and basic solutions. Therefore, a dramatic change of pepsin CSD in ESI-mass spectra acquired under these conditions [76, 77] is observed, and the corresponding most intense CS increased to above +30.

So the analysis of the CSD of proteins in ESI-MS provides reliable information on the protein structure and stability at different solution pH.

### 6.2. Number of Acidic and Basic Functional Groups in the Proteins

Conformation-dependent neutralization theory (CDNT) was proposed to interpret the appearance of main CS in ESI of proteins [83, 84]. The difference between acidic (aspartic acid, glutamic acid, etc.) and basic (arginine, lysine, histidine, etc.) residues in the protein sequence was used to determine the predominant charge state in the ESI-mass spectrum. However, the CDNT could not explain the appearance of the positive ion mass spectra of several proteins although they contain overall negative charge and vice versa. Later it was proposed that only the surface-exposed basic or acidic groups of a protein could hold charge in the gas phase [85]. However, some surface-exposed residues may form salt bridges, ion pair, or hydrogen bonding within the protein, reducing the effective charge density on these residues [86] and thereby decreasing the charge on the protein. Hence, we reported a more refined model to interpret the CS based on the three-dimensional structure (crystal structure) of the protein [78]. In view of this, we have identified the free basic and acidic groups (which do not form any ion pair or salt bridge) on the protein surface from the crystal structure analysis of a series of the proteins. Then we investigated the ESI-mass spectra of those native folded proteins in both positive and negative ion mode [78]. Interestingly it was observed that the maximum charge state of the gaseous protein ion actually corresponds to the number of surface-exposed free basic (in positive ion mode) or free acidic residues (in negative ion mode). This model was further supported by the ESI-mass spectra of the denatured proteins [78]. Upon denaturation all the acidic and basic residues become surface exposed, and the number of the basic and acidic residues in the protein sequence corresponded to the maximum CS in positive and negative ion mode, respectively.

Some exceptional examples of the above model were reported later. One particularly interesting example is the ESI-mass spectra of native folded pepsin (33 kDa) which contains total 41 acidic and only four basic residues [82]. Despite the small number of basic sites, the native pepsin (pH ≤ 2) can accommodate maximum 11 positive charges (most intense CS is +10) in gas phase upon positive ion ESI [76, 77]. This maximum charge state (+11) is higher than that predicted by our model and lower than that predicted by CRM ((4), which predicts +14 as the average CS). Since most proteins possess a high number of basic sites, the requisite number of positive charges as predicted by CRM can almost always be distributed among the available free basic sites on the surface of the native proteins. But, the ability of gaseous pepsin to accommodate a number of protons far exceeding the number of available basic sites might be due to the participation of the other mild basic functional groups (e.g., amide moiety, etc.) or collectively a number of polar functional groups over the large flexible surface of the protein to hold (solvate) protons in gas phase [87]. The lack of the required number of strong basic functional groups in pepsin possibly inhibits the extensive protonation as predicted by CRM. Moreover, it has been proposed that the proton affinity of the solvent molecules provides a “cut-off” level for the amino acid residues that are protonated in gas phase by ESI [87].

### 6.3. Protein Surface Area in Solution

Despite the fact that the extent of multiple charging in ESI is influenced by the number of surface-exposed free acidic/basic functional groups in the proteins (i.e., the gas-phase ion chemistry), the physical dimension or the structural integrity of the protein also largely influence the CSD. Kaltashov and Mohimen [77] demonstrated that the CSD of protein ions in ESI can be correlated to the solvent-exposed surface area in solution. In their study on 22 proteins ranging from the small protein insulin (5 kDa) to the large protein ferritin (500 kDa), they showed that the observed average charge states (*Z*_av_) in the ESI-mass spectra could be correlated more precisely to the solvent exposed surface area rather than the radius or molecular weight of the proteins as suggested by CRM ((3) and (4)). The spherical approximation of the proteins which was used by Fernandez De La Mora [61] could provide erroneous results in CRM ((3) and (4)) since in practice the protein shape can deviate from spherical. Therefore, they extended (3) to (6) as given below to correlate the *Z*_av_ with the protein surface area *S* (Since, *S* = 4*πR* ^2^) [77].

(6) $$Z_{\text{av}}\cdot e=4{\left(4\pi \varepsilon_{0}^{2}\gamma^{2}S^{3}\right)}^{1/4}.$$

**Figure 12:**
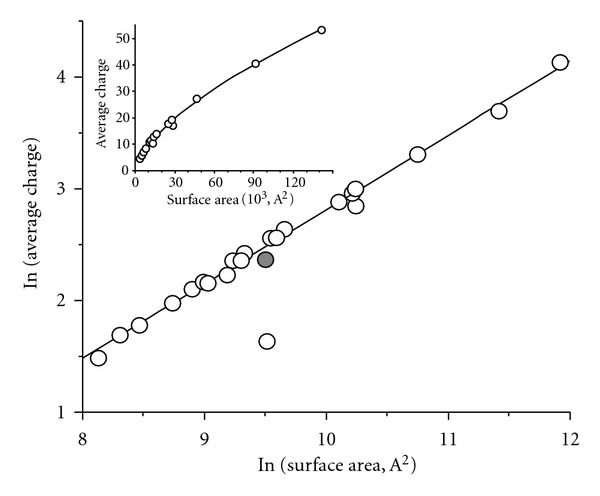
Correlation between the average charge state of protein ions generated by ESI under near-native conditions (10 mM ammonium acetate, pH adjusted to 7) and their surface areas in solution, whose calculation was based upon their crystal structures. The data are plotted in the logarithmic scale (a graph plotted in the normal scale is shown in the inset). A gray-shaded dot represents a pepsin data point. An open circle underneath represents the highest charge of pepsin if the extent of multiple charging was limited by the number of basic residues within the protein molecule. (Reprinted with permission from *Analytical Chemistry *[77], Copyright 2005, American Chemical Society).

Their empirical data is shown in Figure 12, which clearly indicates that the surface area (*S*) evaluated from the known crystal structure and the charge state *Z*_av_, obtained from the ESI-mass spectra of the proteins, are correlated and fitted well by (7) or (8) with the value *a* = 0.69. The fit of the data points (Figure 12) is much better than the fit in the Fernandez De La Mora plot (Figure 8)

(7) $$\begin{array}{cc} Z_{\text{av}} & ={AS}^{a},\\ \ln\;Z_{\text{av}} & =\ln\;A+a\;\ln\;S. \end{array}$$

Thus, they showed that the average CS-molecular mass correlation holds only for the tightly packed globular protein. But, there are some proteins (e.g., ferritin) that are not tightly packed in their native conformations and it is actually the solvent exposed surface area of the proteins that is the major determinant of the average CS of the gaseous proteins produced by ESI process [77].

### 6.4. Presence of Nonvolatile Salts in Protein Solution

**Figure 13:**
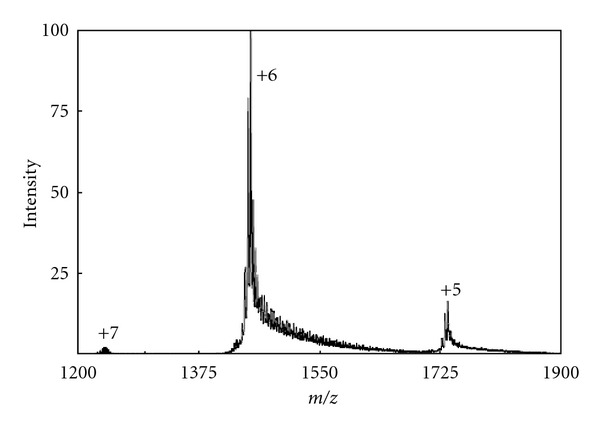
Nano-ESI-mass spectrum of 25 *μ*M ubiquitin in 1 mM NaCl solution. The spectrum was obtained at low nozzle-skimmer potential, so that there was little collisional activation of the protein. It is obvious that each charge state is not a single peak but consists of multiple peaks due to the adduct formation of the salt ions (e.g., Na^+^ & Cl^−^) with ubiquitin. (Reprinted with permission from *Journal of the American Society for Mass Spectrometry* [91], Copyright 2005, Elsevier).

It is often the case that the proteins are purified and stored in some buffer solution containing nonvolatile salts (e.g., sodium chloride, potassium chloride, sodium phosphate, potassium phosphate, etc.). So if the protein solution is not properly desalted before injecting it in the ESI probe, the ions such as Na^+^, K^+^, and so forth, make strong ion pairing with the ionized acidic residues of the protein in the gas phase [88]. The nonvolatile metal ion impurities not only form strong bonds with carboxylate functional groups but also with other polar functional groups including the amide groups in the peptide backbone [89, 90]. This ion pairing would be driven by large loss of the solvent from the droplet and thus concentrating the nonvolatile salt component along with the proteins in the vanishing charged droplet. The binding of those metal ions (Na^+^ and K^+^) with the protein in the gas phase is so strong that they even survive in the clean-up stages (heated capillary, nozzle-skimmer dissociation potential) of the ESI mass spectrometer [91]. But the adduct formation of the metal ions (M^+^) and/or their salts (MA) with the proteins is highly undesirable because of the mass shifting in variable extent that generates multiple peaks corresponding to each charge state (see Figure 13) [91]. The observed multiple peaks corresponding to each charge state can be rationalized by (a) replacement of H^+^ with M^+^ and (b) ion pairing of M^+^ with the ionized acidic residues (aspartic acid/glutamic acid/C-terminal carboxylic acid, etc.) and/or ion pairing of A^−^ (counter anion of the non volatile salt) with the ionized basic residues (Arginine, Lysine, Histidine, N-terminal amine, etc.) [91]. If the mass spectrometer is not of sufficiently high resolution, then a complicated and “messy” spectrum is produced for the adduct formation of the component ions of the salt impurities with the protein in the gas phase.

 In our recent study we have showen that the relative volatility of the charged particles (from salts) and the surface accessibility of the polar residues of the protein towards the charged particles are important factors in the CSD of the protein in gas phase [92]. The surface accessibility of surface-exposed free basic residues (SEFBRs) and surface-exposed free acidic residues (SEFARs) were determined from the crystal structure and compared with the most probable charge-state (most-intense peak obtained in the positive ion and the negative ion mode ESI-MS spectra of the proteins). The results indicated that the number of the SEFBRs that have surface accessibility above the mean surface accessibility of all the SEFBRs in the protein corresponds to the most-intense charge state of the protein in the positive ion mode. Analogously, the number of the SEFARs that have surface accessibility above the mean surface accessibility of all SEFARs in the protein would give the most-intense negative ion charge state of the protein. Hence, the most-intense charge states both in the positive ion as well as in the negative ion modes for a large number of proteins could be predicted based on this simple model from surface accessibility of the protein.

### 6.5. Use of Ammonium Acetate Salt Additive

Ammonium acetate buffer solution is very often used in the mass spectrometric analysis of proteins (see Figure 11) since unlike other buffer of nonvolatile salts it produces very clean mass spectra solely due to the protonated proteins in the gas phase [88, 91]. Even the use of ammonium acetate prevents the formation of sodium/potassium (if present as impurities) adduct with the gaseous protein. When ammonium acetate is added to the protein solution, following ion pairing reaction (8) occurs on the solvent accessed surface of the protein [91]. Ammonium cations pair with acidic functional groups, and acetate anions pair with basic functional groups in the protein in the gas phase upon ESI. The larger concentration of ammonium acetate relative to sodium/potassium ion impurities reduces the possibility of ion pairing involving sodium/potassium because of the predominance of ammonium acetate interaction with proteins (8). When the resulting protein ions containing the ion pairs (8) fly through the heated capillary and nozzle skimmer in the intermediate pressure region of the ESI interface, they encounter collision with the background gas and solvent vapor, which causes loss of volatile acetic acid and ammonia by the proton transfer reaction (9). The process is entropically favorable, and the dissociation is also facile because of the low bond (proton transfer) energy for the dissociation (15 kcal/mol for acetic acid and 11 kcal/mol for ammonia) [91]. The net effect of the complete process is nothing but the proton transfer from the protonated basic residues to deprotonated acidic residues. Though the positive and negative groups are neutralized, the molecular weight of the protein does not change. Thus, a clean mass spectrum of the protein is obtained without the mass change

(8) H3N+-(protein)-COO−+CH3COONH4 =CH3COO−H3N+-(protein)-COO− NH4+,

(9) CH3COO−H3N+-(protein)-COO− NH4+   =CH3COOH + H2N-(protein)-COOH + NH3.

Felitsyn et al. [93] and Peschke et al. [94] also suggested that, in presence of the volatile salt like ammonium acetate, the ammonium cations can also bind to the basic residues of the protein, and there is proton transfer reaction from the ammonium ion to the basic residues of the protein with formation of volatile ammonia (base) and the protonated protein in the gas phase. Thus, ammonium acetate can also enhance the propensity of protonation of the protein in the gas phase.

The above equations ((8) and (9)) suggest that the use of ammonium acetate not only suppresses the metal ion adduct formation but also prevents the adduct formation involving the anion of strong acids (e.g., phosphate, trifluoroacetate anions, etc.) and ionized basic residues. Though (trifluoroacetic acid) TFA is frequently used in the mobile phase of the liquid chromatography mass spectrometry (LC-MS), the presence of the TFA anion in the effluent causes the loss of sensitivity, and the use of ammonium acetate prevents that sensitivity loss [95].

### 6.6. The Nature of Solvent

Several physical properties of the solvent like polarity (dielectric constant) [96], gas-phase basicity [97–99], and the surface tension [61, 100, 101] are also known to be responsible for the CSD of the protein. It is expected that the solvent with higher dielectric constants will tolerate high charge densities in the evaporating droplets produced by the electrospray process. The excess charges can also be confined to a thin layer at the surface because of the increased conductivity of the polar solvent with higher dielectric constant. At the last stage of solvent evaporation or gaseous ion emission from the charged droplet, high polarity solvents are more effective in stabilizing the multiply charged species, whereas the low polarity solvent may disfavor that. So the solvent with higher dielectric constant would boost higher charge states in the gaseous analyte ions generated from those ES solvent droplets. Loo et al. also showed that the denaturing capacities of different solvents also affect the protein CSD, which in turn reflect the conformation (solvent accessibility) of the protein in that solution [102].

**Figure 14:**
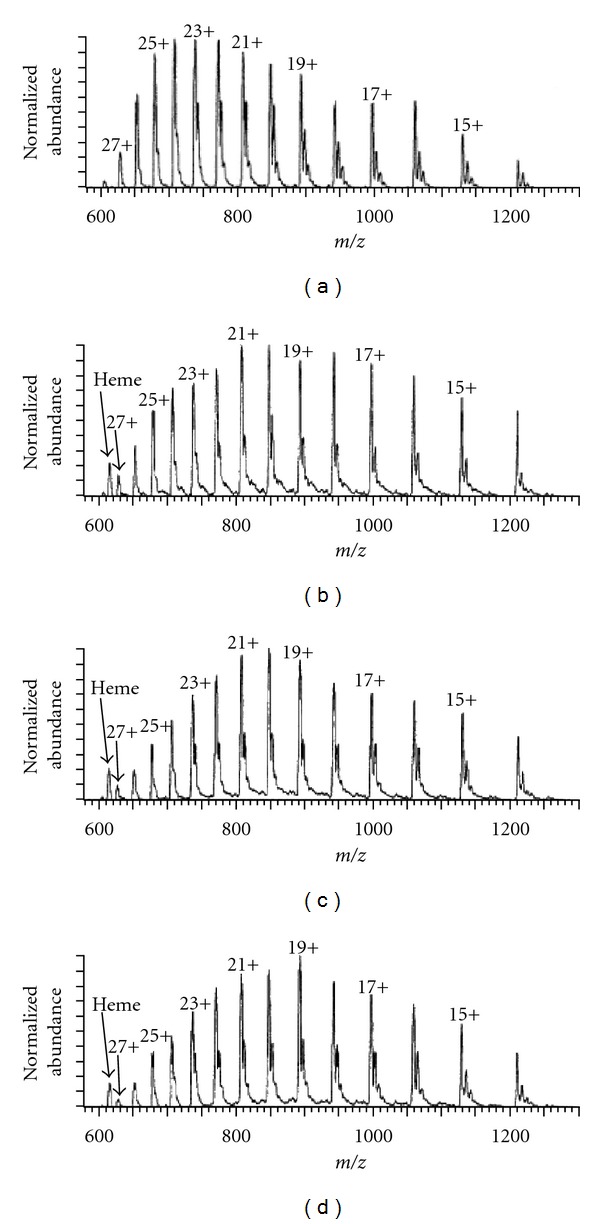
Myoglobin (10^−5^ M) electrosprayed from 47%/50%/3% water/solvent/acetic acid solutions, where the “solvent” was (a) water, (b) methanol, (c) acetonitrile, or (d) isopropanol. (Reprinted with permission from *Journal of the American Society for Mass Spectrometry* [96], Copyright 2000, Elsevier).

Apart from the apparent gas-phase basicities (calculated based on the intrinsic gas-phase basicities and Coulomb energy of the interacting charges) of the basic sites in the protein, the gas-phase basicity of the solvent is also an important factor to significantly affect the CSD of the protein [87, 103]. The effect of the solvent composition on both maximum CS and the CSD of the proteins were investigated by Iavarone and coworkers [96]. They showed that CSD of cytochrome c and myoglobin (see Figure 14), formed from 47%/50%/3% water/solvent/acetic acid solutions, shifts to lower charge (higher *m/z*) when the 50% solvent fraction is changed from water to methanol, to acetonitrile, to isopropanol. This is also the order of increasing gas-phase basicities of these solvents. However, the effect is relatively small for these solvents, possibly due to their limited concentration (in gas phase) inside the electrospray interface. But the addition of small amounts of diethylamine (0.4%) in the aspirating solution results in dramatic shifts to lower charge, presumably due to preferential proton transfer from the higher charge state ions to diethylamine. These results clearly show that the maximum charge states and charge state distributions of ions formed by electrospray ionization are influenced by solvents that are more volatile than water and the gas-phase basicity of the solvent too [96].

It is also not surprising that along with different physical properties of the solvent the surface tension plays an important role to the CSD of the protein, since the surface tension parameter (*γ*) is involved in the Rayleigh equation (3) [62], based on which the CRM is proposed [61]. The increase in surface tension of the solvent should increase the average charge state of the analyte. Iavarone and Williams showed that, in absence of other factors, the surface tension of the ultimate electrospray droplet (formed after repeated solvent evaporation and Coulomb fission), from which the charged gaseous analyte is formed, is a significant factor in determining the overall analyte charge [100].

### 6.7. The Supercharging Effect

**Figure 15:**
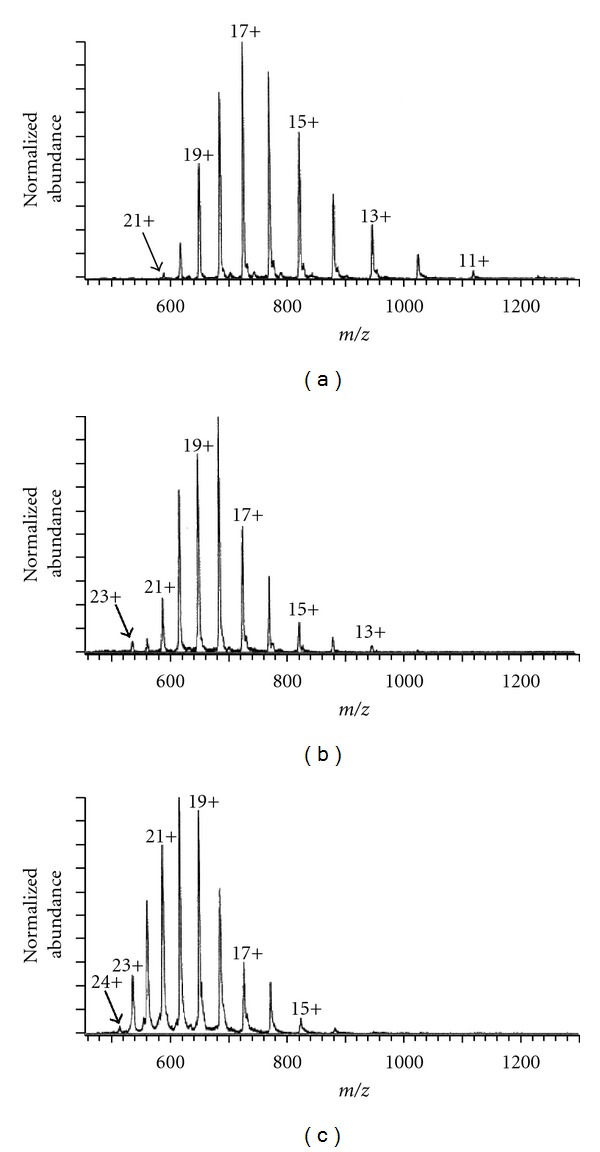
Electrospray ionization mass spectra of cytochrome c (10^−5^ M) from solutions containing (a) 0, (b) 0.3, and (c) 0.7% m-NBA. The base solution is 47/50/3% water/methanol/acetic acid. (Reprinted with permission from *Analytical Chemistry* [101], Copyright 2001, American Chemical Society).

Iavarone and coworkers reported interesting additives, for example, m-nitrobenzyl alcohol (m-NBA) and glycerol to the aqueous solution of the protein and peptide, which can dramatically increase both the maximum charge state and the most intense charge state [101]. They termed this phenomenon “supercharging effect.” Figure 15 shows that the addition of just <1% m-NBA to the electrospray solution, the maximum charge state as well as average charge state increases significantly [101]. Similarly the addition of small amount of m-NBA in the peptide (KKKK) solution substantially increased the most intense and average CS [101]. The exact mechanism of this enhanced charging is still not clear. Recently they have suggested that these supercharging reagents have low vapor pressures (less volatile), and aqueous droplets are preferentially enriched in these reagents as evaporation occurs [100]. Less evaporative cooling will occur after the droplets are substantially enriched in the low volatility supercharging reagent, and the droplet temperature should be higher compared with when these reagents are not present. Protein unfolding induced by chemical and/or thermal denaturation in the final electrospray droplet, from which the charged gaseous protein is produced, appears to be the primary origin of the enhanced charging [104, 105]. The fact is further supported by the arrival time distributions obtained from traveling wave ion mobility spectrometry [105], which showed that the higher charge state ions that are formed with the supercharging reagents are significantly more unfolded than lower charge state ions. Though this model successfully explained the supercharging of proteins and their noncovalent complexes, it fails to explain the supercharging of small molecules like small peptides [101], which do not have any tertiary structure. A detail investigation of the physical parameters of the evaporating and disintegrating droplet containing this supercharging reagent is required in the future to understand the exact mechanism of supercharging.

Recently Lomeli group has reported other supercharging agents, for example, benzyl alcohol, m-nitroacetophenone, m-nitrobenzonitrile, o-NBA, m-NBA, p-NBA, m-nitrophenyl ethanol, sulfolane (tetramethylene sulfone), and m-(trifluoromethyl)-benzyl alcohol [106]. Among these sulfolane displayed a greater charge increase (61%) than m-NBA (21%) for myoglobin in aqueous solutions based on average charge state. All these reagents that promote higher ESI charging appear to have low solution-phase basicities and relatively low gas-phase basicities and are less volatile than water [106].

Thus the increased multiple charging by the supercharging reagents promises its implication in the accurate mass measurement of the large macromolecules and the structural characterization of the proteins by tandem mass spectrometry and MS^*n*^ experiments, where the multiple charging is important for efficient fragmentation (discussed later) [107–110].

### 6.8. Modification of the Protein Charge State Distribution

Several approaches like acid/alcohol-induced proteins denaturation [102], protein disulphide bond reduction using chemical reducing agent dithiothreitol, and so forth, [50] were undertaken to modify the protein CSD in last two decades. When the disulfide bonds are reduced, much higher charge states can be obtained because the reduction of the disulfide bonds allows the protein to unfold further and expose additional acidic/basic sites for protonation/deprotonation [50].

Though the protein CSD can be altered by manipulating the solution conditions (pH, reduction of disulphide bonds, use of different solvents, denaturing agent and supercharging agent, etc.), it can also lead to deleterious effects such as poor ESI response, dissociation of noncovalent complexes, and so forth. But, sometimes it is desirable to alter CSDs independent of initial solution conditions. The CS alteration by gas-phase proton transfer reaction (post-ESI process) has been demonstrated via ion/ion [111, 112] or ion/molecule [113] reaction. But that resulted in significant reduction of the absolute charge of the gaseous analytes. Recently Kharlamova and coworkers have established a method for the manipulation of the CSD of the electrosprayed proteins by exposing the nano ES droplets to the volatile acid/base vapors in the atmospheric pressure region of the mass spectrometer [114, 115]. Exposure of the positive ES droplets to the acid vapors [115] and the negative ES droplets to the base vapors [114] increases the propensity of high CS formation of the analyte (protein). On the basis of changes in protein CSDs, protein folding and unfolding phenomena are implicated in their studies. Since no change in the CS distributions of buffered proteins exposed to reagent acid/base vapors was observed, the charge state changes are attributed largely to a pH effect. They suggested that, when the acid/base vapor interacts with the nanoelectrospray generated protein droplets, it changes the pH of the droplets and thereby affects the structure of the protein present in the ES droplets during the very short nanodroplet lifetime (few tens of microseconds). This change of the protein structure is attributed by the change of the CSD. They also showed that the species bound by relatively weak interactions can be preserved, at least to some extent, allowing for the observation of high charge states of protein-ligand complexes [114, 115].

Recently Krusemark et al. used chemical derivatization method to control the CSD, which eventually gave the answer about the role of protein functional groups in the CSD [116]. When the free carboxylic acid function groups of the denatured proteins were modified by amidation (neutral functional group), minimal change in the CSD in positive ion mode was observed compared to the unmodified proteins, indicating that the carboxylic acid functional groups do not play significant role in charging in the positive ion mode. The modification of proteins with additional basic sites or fixed positive charges generated substantially higher charge states in positive ion mode, providing evidence that the number of ionizable (basic) sites determines ESI charging for denatured proteins irrespective of their shape and sizes. Combining this chemical modification and the ion/molecule or ion/ion reactions in gas phase, the authors also illustrated some unique approaches to alter/control the CSD [117].

### 6.9. Analyte Concentration and the Presence of Electrolyte Impurities

Apart from the analyte structure and conformation, the analyte concentration in the initial spray solution is also an important factor that significantly affects the ESI-MS signal intensity [22, 39, 118]. For multiply charged analyte, ions of discrete charge state exhibit a linear response of the ion signal with the analyte concentration in solution until a certain concentration limit is exceeded. This concentration limit is dependent on the nature of the analyte. Beyond that concentration the signal intensity loses that linear response and may even decrease at extremely high concentration. This departure from the linearity as the aspirating solution concentration increases can be attributed to the analyte concentration having reached its saturation value during the droplet evaporation so that further loss of solvent results in precipitation, rather than any further increase in its concentration in solution [39]. At elevated concentration the relative abundances of the gaseous analyte ions of higher charge state also decrease due to the increase in competition between the analyte molecules inside the ES droplet for the limited number of available charges on the droplet surfaces [119].

 The presence of any ionic species (i.e., electrolytes) other than analyte in the solution (except for small amounts of electrospray friendly acids, bases, or buffers as discussed before) is usually avoided when possible, because their presence in the ES solution has been found to suppress the formation of gas-phase ions of the analyte of interest [118, 120–122].

### 6.10. Instrumental Parameters

Very often it is observed that the CSD in the ESI-mass spectra of a particular analyte acquired from different instruments is significantly different though a specific protocol for the sample preparation has been followed. Moreover, ES-mass spectra acquired from the same instrument at different points in time can show some degree of variability. This is because there are several instrumental parameters which are responsible for the ion production, filtration, and detection. The instrumental parameters like spray tip orifice diameter (e.g., microspray and nanospray [32, 123]), ion spray voltage (different onset voltages for different solvents [22]), source geometry [98, 124] (e.g., on-axis, off-axis, etc., [125]), source gas pressure [39, 126], source temperature [58], heated capillary temperature (thermal dissociation may occur here) [58, 126–128], sheath/auxiliary gas flow rate [39] that (affects the evaporation and cooling rate of the ES droplets), nozzle-skimmer voltage (declustering potential) [129–132], different ion-optics voltages [34, 58] (that guide the ion filtration and trapping) [34, 58] and nature of the detectors (detector response) [34, 58] are all known to influence the ESI-MS response of the analyte.

## 7. Is Substantial Conformational Change of the Protein Possible inside the ES Droplet?

There are still several aspects of ESI not fully understood though it is almost two and half decades elapsed after the discovery of ESI-MS. One such major issue is the conformational and other subtle chemical properties of the analyte during the transfer from solution to gas phase. The issue becomes further complicated due to the complexity of the processes affecting the ES droplets including the charge separation [22], solvent electrolysis at the emitter tip [133, 134], droplet evaporation then subdivision [22] the increase of the droplet acidity/basicity, and so forth. As discussed earlier the droplets produced at low flow rate (typically ~5 *μ*L/min) have a narrow distribution of sizes with a most abundant radius ~1.5 *μ*m [22]. Such an electrospray droplet was earlier shown to have a charge of ~10^−14^ C, which corresponds to ~60,000 singly charged ions [16]. If we assume that all these singly charged ions are protons (in positive ion mode) and they are homogeneously distributed in the droplet, then the estimated pH of the initially produced droplet would be ~5.2. The repeated solvent evaporation and Coulomb fission of the droplets would increase the proton density and thereby continuously lower the pH of the droplets. According to the IEM, the gas-phase ions are supposed to be formed when the droplet reaches the radius ≤10 nm [63]. The Rayleigh charging [62] at this limit (*r* = 10 nm) corresponds to 125 elementary charges (protons) in the droplet. So the corresponding pH of the droplet at this limit is ~1.3. The acidity of the ES droplets was also experimentally measured [135]. Overall the chemical composition in the final small droplets from which the naked analyte ions are formed may be significantly different from that of the original sample solution [22]. For example, the acid/base equilibrium, the conformational states, and/or noncovalent bonding in the analyte may all be affected by the subtle changes in the composition and properties of the droplets during the electrospray. There have been no reports addressing the possible effects of the microenvironment of the solvent droplets on the analyte during the ES process. Generally it has been assumed that the solute molecules residing at the neutral core of the charged droplet remain “oblivious” to the harsh conditions on the droplet surface and the environment beyond it until the last of the solvent molecules is gone. As a consequence it is presumed that the protein charge state distribution in the gas phase actually represents the protein conformation in the initial solution. In some reports it has been also assumed that the violent nature of charged jet breakup and subsequent Coulombic explosion of the ES droplets encapsulating the proteins can cause repacking of the supramolecular assemblies, forcing them to hold a minimal volume [77, 136]. But no direct experimental evidence is still available to support this assumption on the molecular rearrangement inside the charged ES droplets. So the molecular behavior inside the charged ES droplets still remains like a “black box” and the exact processes that happen to a molecule residing inside the charged droplets are far from being understood.

If the physical and chemical properties of the ES droplet continuously change during the lifetime of the droplet then what happens to the conformation of the proteins residing inside the charged droplet? Does a protein change its tertiary structure or conformation inside the violently behaved (charged jet breakup) charged ES droplet? The preservation of the overall protein integrity via ES process does not necessarily mean the retention of the initial solution structure intact inside the charged droplets. Some recent works [137, 138] suggested that after the ES process the new gaseous environment can ultimately cause dramatic structural alteration of the protein (post-ESI process) and the existence of gaseous macromolecules (proteins) with a number of different conformations [138]. Though in the solvent-free environment the protein conformational change is not surprising, but the issue regarding the conformational change inside the ES droplet relative to the initial solvent is still far from being understood. If the protein molecule is susceptible to change their three-dimensional conformation inside the ES droplet, then the CSD in the corresponding ESI-mass spectrum should obviously reflect the three-dimensional structure of the protein inside the droplet not in the initial solution.

**Scheme 1:**
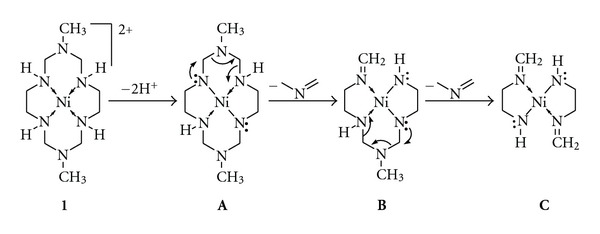
The fragmentation pathway of the cationic complex **1 **inside the charged droplet.

In view of this we have recently conducted some experiments [139] on some model analytes (small molecules) that are labile to be transformed inside the charge droplet. It is often argued that the charges on an ES droplet are distributed over its surface with equidistant spacing and that they are locked into this pattern by the forces of Coulomb repulsion [39], and the bulk or core of the ES droplet essentially remains free of charge to minimize the potential energy of the droplet [140]. But when we put a highly stable intrinsically charged molecule inside the charged ES droplets, we observed a drastic transformation of their structure due to huge Coulomb force of repulsion on the positively charged analyte imparted by the surface protons of the ES droplet (see Scheme 1). This result unambiguously suggests that the violent nature of the charged ES droplets can affect the analyte residing inside it depending on the nature of the analyte [139].

Recently we have also induced the protein conformational change inside the charge droplet by perturbing the droplet solvent composition during the lifetime of the droplet (unpublished work). But it is not clear to us whether without perturbing the solvent composition of the charge droplet the protein conformational change is possible or not in the natural way of ES process.

## 8. Tandem Mass Spectrometry: Collision-Induced Dissociation

The word “tandem” means arrangement of two or more objects/persons one behind another, [141]. Similarly tandem mass spectrometry (MS/MS) is a method where the gaseous ions are subjected to two or more sequential stages of mass analysis (which may be separated spatially or temporally) according to quotient mass/charge [40, 142]. For example, in a tandem mass spectrometric experiment, a precursor ion is mass selected by a mass analyzer Q_1_ (see Figure 4(b)) and then focused into a reaction cell q_2_ (collision cell) where it undergoes a gas-phase chemical reaction. The reaction gives different product ions with different masses, which are then passed to another mass analyzer Q_3_ (see Figure 4(b)). This last mass analyzer scans the masses of the product ions and generates the product ion spectrum. The mass analyzers are set up in series either in space (sector, triple quadrupole, and hybrid instruments) or in time (trapping instruments).

ESI transforms the analyte in gas phase without rupturing any covalent bonds and provides the information about the molecular weight (MW). This MW alone can not determine the structure of an unknown analyte. So, the fragmentation of the gaseous analyte is deliberately required for the structural elucidation. The fragment ion spectrum is basically the fingerprint of the presence of a particular analyte (precursor ion). By investigating the fragmentation reaction (fragment-ion spectrum), one can actually determine the correct chemical structure of the unknown analyte sample. The task is like a jigsaw puzzle, where the players have been given a broken plate and asked to join them together in a sensible way to find the actual shape of the plate. Now the question is how one can fragment a charged molecule in gas phase? The mass-selected ion (precursor analyte) is activated in the collision cell (e.g., q_2_ in Figure 4(b)) by increasing its internal energy. This activation causes the homolytic or heterolytic cleavage of the chemical bonds. As a result different fragment ions (product ions) are produced. There are different methods of precursor ion activation, which include collisional activation (collision-induced dissociation or CID [143], surface-induced dissociation or SID [144, 145], etc.), photon-induced activation (infrared multiphoton dissociation or IRMPD [146], blackbody infrared radiative dissociation or BIRD [147, 148], ultraviolet photodissociation or UVPD [149, 150], etc.) and electron-mediated activation (electron capture dissociation or ECD [151], electron transfer dissociation or ETD [152], electron detachment dissociation or EDD [153], electron-induced dissociation or EID [154]), and so forth. Here we would discuss about the most popular and most common ion activation method in the ESI-MS system, which is collision-induced dissociation (CID).

In collision-induced dissociation, the gaseous precursor ion is allowed to collide with a gaseous target in the collision cell. The gaseous target is inert and neutral, generally inert gases such as nitrogen, helium, or argon [155]. As a result of collision, the energy is gained and redistributed among different vibrational degrees of freedom (internal energy) within the precursor ion. Thus, an unstable excited state of the precursor ion is populated, which causes the precursor ion to decompose into the product ions in a process termed “collision-induced dissociation” (CID) [143]. So the CID is basically a unimolecular fragmentation reaction of the mass-selected precursor ion.

### 8.1. Multistage CID/Tandem Mass Spectrometry

But it is often the case that the single-stage collision-induced dissociation provides a lot of confusing fragmentation information or does not provide enough information for the structural elucidation. In both of these cases multistage tandem mass spectrometry (MS*^n^*)/multistage CID is performed. Generally MS*^n^* experiments are performed in ion-trap or in FT-ICR instrument, where each MS step is separated in time [58]. Those trapping instruments allow the refragmentation of the product ions and thus produce next generation of product ions and so on. For example, in the first stage, the normal ESI-mass spectrum (MS^1^) is produced without fragmentation. Then one precursor ion is isolated in the trap and fragmented to produce the first generation product/fragment ions, and the corresponding spectrum is called MS/MS or MS^2^ spectrum. Again one precursor is isolated from the first generation fragment ion (from MS^2^ spectrum) and then fragmented to produce the second generation product ions, which are scanned to record the MS^3^ spectrum (MS/MS/MS). Thus process of isolation and fragmentation can be repeated a number of times, resulting in a series of MS*^n^* spectra where “*n*” represents the number of times, that is, (*n* − 1) times, the isolation-fragmentation cycle has been carried out.

### 8.2. High- and Low-Energy CID

The appearance of the CID spectrum is dependent on several parameters like nature of the projectile ion and target gas, target gas number density, ion activation time, nature of the collision cell, and above all on the collision energy between gaseous projectile ion and the target atom/molecule [58]. This collision is controlled by the kinetic/translational energy of the projectile. The fraction of the kinetic energy that can be converted to the internal energy of the precursor ion in a collision is determined by the centre of mass collision energy (*E*_cm_) [143]

(10) $$E_{\text{cm}}=\frac{m}{m+M}E_{K},$$

where “*m*” is the atomic/molecular weight of the target gas “*M*” is the molecular weight of the precursor ion, and “*E*_K_” is the precursor ion kinetic energy.  *E*_cm_ is the maximum amount of the precursor ion's kinetic energy, which can be converted into the internal energy for fragmentation. The *E*_cm_ above 25 eV is defined as high collision energy and that below 20 eV as low collision energy [58, 158, 159]. Since a very high kinetic energy (KeV) is gained by the projectile ion in the traditional sector instruments, the high energy CID spectrum is produced in those instruments [58]. But the projectile translational energy is in the range tens to hundreds of electronvolts in the triple-quadrupole instruments and thus those instruments provide low energy CID spectrum [58]. In the ion-trapping instruments (e.g., QIT), the ions are trapped within a three-dimensional electric field inside the ion trap, and a stable ion trajectory is guided by Mathieu equation. So in QIT the precursor ion kinetic energy can only be raised to a level where stable ion motion still occurs. Consequently the achievable kinetic energy of the precursor ion falls in the range of low energy CID regime [58].

 In low energy CID, the internal energy increase is sufficient to vibrationally (not electronically) excite the precursor ion, which results in the cleavage of the most labile bonds when a certain threshold energy is reached. But in high energy CID, electronic excitation of the precursor ion becomes possible if the ion-neutral interaction time is very short (<10^−14 ^s). But this generally does not occur for medium-to-large size molecules undergoing high-energy CID [58]. In those cases a curve-crossing mechanism more likely occurs [58]. For example, a crossover from the first electronically excited state to the higher vibrational level of the ground state occurs. But both direct electronic excitation and curve-crossing are likely to result, a different fragmentation pattern compared to that in the low-energy CID. Although the high-energy CID spectrum is typically much more complex than low-energy spectrum, it contains maximum structural information. For example, generally backbone cleavage of the peptide is observed upon low-energy CID but side-chain cleavage including, backbone cleavage of the peptide is observed upon high-energy CID [159–161]. High-energy CID is known to promote charge-remote fragmentation [162, 163], while low-energy CID is often guided by charge-directed fragmentation mechanism (see later) [161].

 Here we would discuss the low- and/or high-energy fragmentation of some biologically important molecules like peptides, oligosaccharides, oligonucleotides, and lipids, which are very often analyzed by ESI-CID-MS/MS experiments.

### 8.3. CID of Peptides

**Figure 16:**
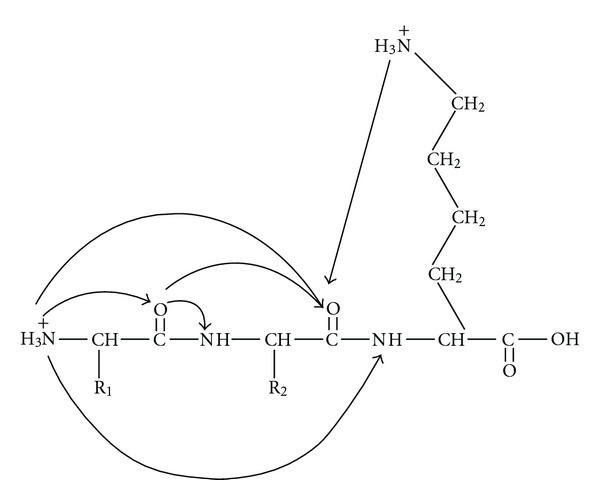
Mobile proton model. (Reprinted with permission from *Journal of the American Society for Mass Spectrometry* [156], Copyright 2010, Elsevier).

**Figure 17:**
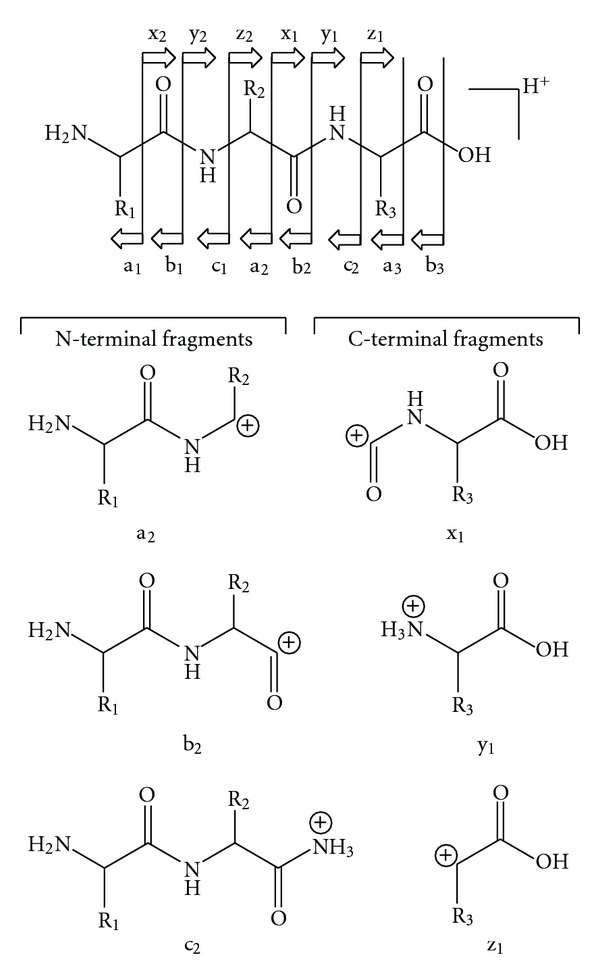
Nomenclature of the peptide fragments showing some typical qualitative structure of the fragments.

**Figure 18:**
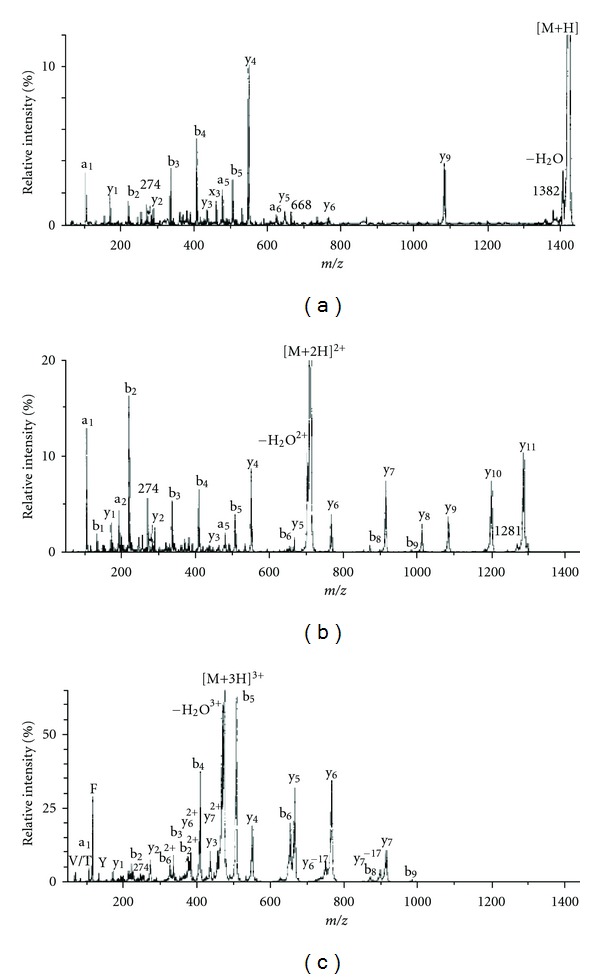
CID-MS/MS spectra of different protonated forms of the peptide HSDAVFTDNYTR: (a) CID of singly protonated peptide [M + H]^+^, (b) CID of doubly protonated peptide [M + 2H]^2+^, and (c) CID of triply protonated peptide [M + 3H]^3+^. (Reprinted with permission from *Analytical Chemistry* [157], Copyright 1993, American Chemical Society).

When a peptide is electrosprayed in positive ion mode, gaseous protonated peptide is produced. The protons are mostly localized on the most basic sites (e.g., Arg, Lys, and his side-chain or N-terminal *α*-NH_2_ group) in the peptides prior to activation [164, 165]. Upon collisional activation, the ionizing protons are transferred from the unreactive sites of higher gas-phase basicity (e.g., Arg, Lys, and his side-chain or N-terminal *α*-NH_2_ group) to several backbone amides to form energetically less favored but reactive backbone-amide-protonated species [161]. The amount of activation energy that has to supply to the precursor-protonated peptide to make the proton sufficiently mobile is dependent on the gas-phase proton affinities of the different amino acids (Arg > Lys > His > Try > Glu > Pro > Gln > Met > Tyr > Asn > Phe > Thr > Ile > Leu > Val > Asp > Ser > Ala > Cys > Gly) [166, 167] present in the sequence. For example, when a doubly protonated peptide, which contains a strongly basic amino acid residue (e.g., Arg/Lys/His), is collisionally activated one proton would mostly be anchored on the basic residue, and the second one would be mobile in the backbone leading to the heterogeneous population of the ions varying the location of the second proton. This fast proton “dancing”/migration process to various backbone amide bonds is called “mobile proton model” as proposed by Wysocki (see Figure 16) [156, 168, 169]. This mobile proton model has been verified by hydrogen/deuterium scrambling studies [170] and theoretically also [171, 172]. Since amide nitrogen protonation removes the resonance stabilization of the amide bond, the protonated amide bonds become weak and labile to fragment in low-energy CID. As a result of heterogeneous population of the activated precursor ions, a series of peptide bonds are cleaved [161, 173]. Apart from the amide bond fragmentation, other backbone bond fragmentation is also possible via mobile proton model. A notation has been developed to indicate the peptide fragments that arise from the CID of the protonated peptides. The N-terminal fragment ions (charge is retained on the N-terminus) are indicated by a_*n*_, b_*n*_, and c_*n*_, and C-terminal fragment ions (charge is retained on the C-terminus) are indicated by x_*n*_, y_*n*_, and z_*n*_ [174, 175] (see Figure 17). The subscript “*n*” indicates the number of amino acid residues in the fragment. As shown in Figure 17, the N-terminal a_*n*_, b_*n*_, and c_*n*_ ions are complimentary to the C-terminal x*_(n−m)_*, y*_(n−m)_*, and z*_(n−m)_* ions, respectively, where “*m*” is the total number of constituent amino acids in the precursor peptide. Under low-energy collision condition, the peptide dissociation mainly leads to the formation of N-terminal a, b-fragments, and C-terminal y-fragment [58, 161]. As a qualitative basis, the fragment with higher gas-phase basicity (GB) will remain protonated with higher probability than the fragment with lower GB [161]. New fragment can further be created, under multiple collision condition, by the primary fragment dissociation leading to the secondary ion products (satellites) such as internal fragments [176], amino acid-specific immonium ions [177], neutral-loss a/b/y-ions (e.g., b_*n*_-NH_3_, b_*n*_-H_2_O, etc.) [178, 179], and smaller member of b, a, and y-ion series. All these ions provide peptide sequence information directly and that is why they are called “direct sequence ion” [180].  Figure 18 shows a typical example of the CID-MS/MS spectra of the different protonated forms of the peptide HSDAVFTDNYTR representing different “direct sequence ions” [157].

**Scheme 2:**
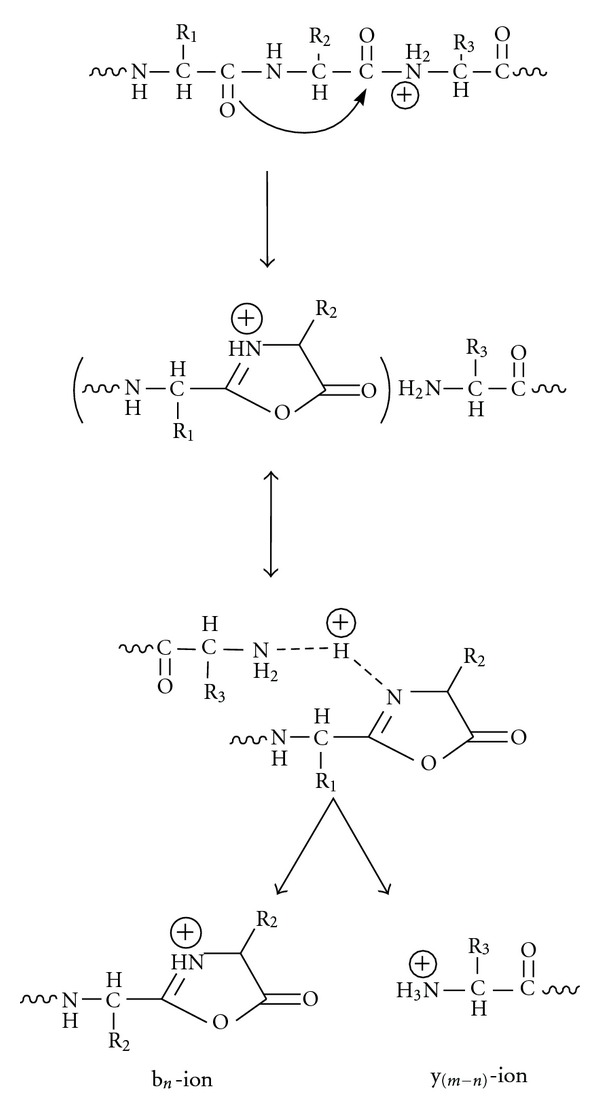
A mechanism for the production of b*_n_* and y*_n_* ions.

 The actual mechanism by which the peptide backbone is cleaved follows complicated pathways. Although the peptide fragment ions (product ions) were identified long ago [107, 174], it is the recent trend to understand the mechanism of the fragmentation of gaseous protonated peptides. For example, recently it has been understood how a singly protonated peptide can fragment to produce the C-terminal/N-terminal fragment ions [181]. The b_*n*_-ion does not exist as acylium cation as shown in Figure 17, rather they form a cyclic oxazolone structure in the gas phase.  Scheme 2 shows the proposed mechanism of the amide bond cleavage of a singly protonated peptide [181]. Before the cleavage of a protonated amide bond, the corresponding amide carbonyl is attacked by the nucleophilic oxygen of the previous amide carbonyl group and thus makes an oxazolone ring followed by a proton-bound complex between the oxazolone (N-terminal) and a truncated peptide or amino acid (C-terminal). Then the fragmentation of the proton-bound complex can occur by the proton transfer process leading to either b_*n*_-ion or y_*n*_-ion. Thus, the propensity of the formation of b_*n*_ or y_*n*_ ion is dependent on the relative proton affinities of the N-terminal oxazolone and the C-terminal truncated peptide/amino acid. The N-terminal oxazolone (b_*n*_-ion) can undergo a neutral loss of carbon monoxide (CO) to form a_*n*_-ion. It has also been proposed that the N-terminal a, b-fragments are terminated with five membered [182] (oxazolone) ring, via rearrangement type reaction. Generally the amide bonds N-terminal to proline and C-terminal to Asp/Glu are more prone to fragmentation [183]. This is because the tertiary amide nitrogen of the proline residue is more basic and thus more likely to be protonated than other backbone amide bonds due to the mobile proton mechanism and thereby leading to enhanced peptide bond cleavage. The acidic residues Asp/Glu provide “local” mobile proton, which catalyzes the cleavage at the peptide bonds C-terminal to these residues [183]. It should be noted that the dissociation of the doubly or multiply charged peptide is more advantageous compared to that of a singly protonated peptide [58]. The population of more than one charge on the peptide backbone results in more dissociation pathways by mobile proton mechanism and thus produces more fragment ions to provide much more sequence information compared to that derived from the singly protonated peptide. Furthermore, the presence of more than one charge of similar polarity can impart Coulomb repulsion in gas phase rendering the precursor ion less stable and thereby further contributes to more facile dissociation. In general the peptide fragmentation is mostly influenced by the charge (e.g., protons), and thereby the fragmentation is called “charge directed fragmentation” [159, 161].

 However, this conventional knowledge of fragmentation pathways and rules cannot reasonably explain many anomalous fragmentations because it is often the case [157, 184–187] that abundant fragment ions appear in the MS/MS spectra of peptides that do not belong to the above-discussed direct sequence ion series. These fragments are formed in complex rearrangements and are termed as “nondirect sequence ions” [180]. The nondirect sequence ions discovered so far include scrambled fragments of the b/a type ions, which is equivalent to the loss of amino acid residue from the interior of the peptide chain [180]. As reported in the recent literatures [180, 187–192], the first step of the mechanism concerned to the internal amino acid loss (sequence-scrambling fragmentation pathway) is a nucleophilic attack by N-terminal *α*-NH_2_ group on the acylium carbocation of the b-ion (a primary fragment) forming a protonated cyclic-peptide intermediate (CPI). This CPI reopens at other sites, producing linear fragment ions where residues are relocated at the C/N-termini. These linear fragment ions further dissociate via conventional fragmentation pathways, forming daughter ions with neutral losses of internal residues from the parent ions. These types of ions are called the nondirect sequence ions of the protonated parent peptides. At the first stage of CID, this unique fragmentation of singly charged b-ions is difficult to be observed. However, under multi-stage CID, almost all of the investigated b-ions (containing three to seven residues) notably displayed the above unique fragmentation patterns. The propensity of the sequence scrambling increases with the increase in the chain length and the charge states of the peptides [193]. A recent report [194], on the basis of the analysis of 15,897 low-energy and 10,878 high-energy CID mass spectra of doubly protonated tryptic peptides, is suggesting that the rate of sequence scrambling due to b-ion cyclization is negligible (<1%) and can be safely ignored as a possible source of erroneous sequence assignment in shotgun proteomics. Recently we have also found the loss of a specific amino acid residue from the interior of the protonated peptide in gas phase, which follows a degradation mechanism other than the above sequence scrambling pathway (unpublished work).

 Although historically the peptide fragmentation is performed on the gaseous protonated peptides to derive the sequence information [173], fewer efforts have been invested on the dissociation of the gaseous deprotonated peptides. Hence the dissociation pathways of the deprotonated peptides are not well established [173]. It should be noted that in negative ion mode the –OH functional groups in serine, threonine, and tyrosine side-chains and –COOH functional groups in glutamic and aspartic acid side-chains are the potential deprotonation sites [195, 196]. Fragmentation of deprotonated peptides has been shown to provide complementary structural information to their positive counterparts [196–198]. Therefore, sequencing unknown peptides can benefit from the interpretation of both positive and negative mass spectra.

 Several aspects of CID of the protonated peptides are still under controversy. It is continuously being debated where exactly the proton is located before the amide bond cleavage, whether the location is carbonyl oxygen or amide nitrogen [107, 164, 168, 171, 199, 200]. How exactly the fragmentation pattern is dependent on the amino acid sequence of the precursor peptides and how different fragments get stabilized? Apart from sequence informative ions, there are lots of anomalous fragments, which are observed in the MS/MS spectra of the peptides. As a future outlook the detail understanding of the formation of those anomalous fragment ions is required and that continuously drawing the attention of the mass spectroscopists.

### 8.4. CID of Oligosaccharides

**Figure 19:**
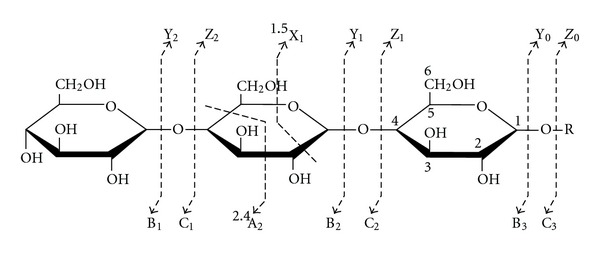
Nomenclature of the product ions formed from the precursor oligosaccharide.

**Scheme 3:**
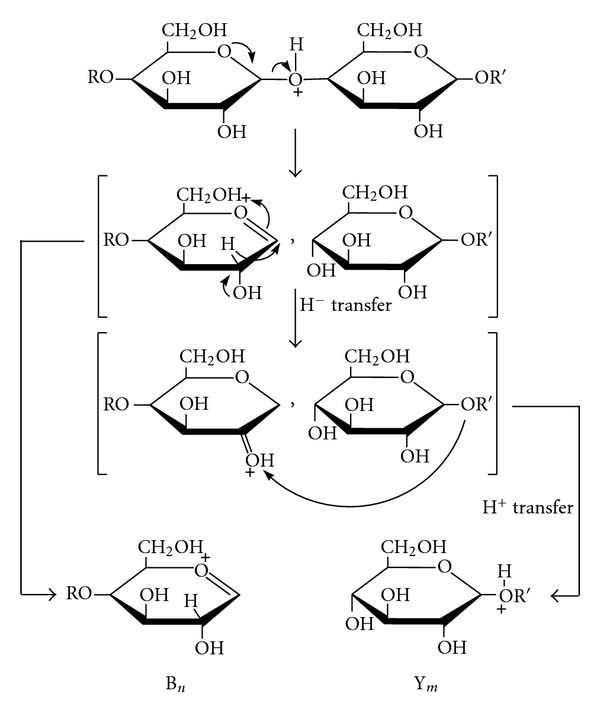
The proposed mechanism of the formation of B*_n_* and Y*_m_* product ions via ion-dipole complex.

**Scheme 4:**
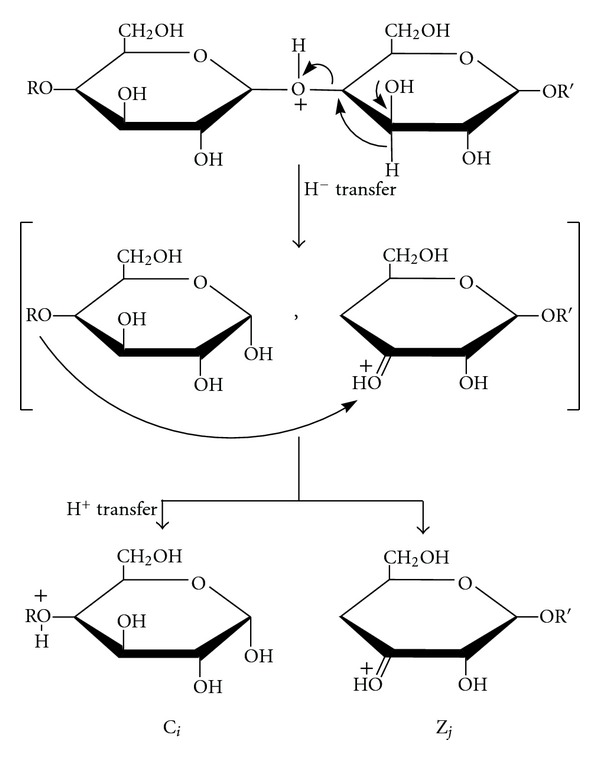
Proposed mechanism of the formation of C*_i_* & Z*_j_* product ions via ion-dipole complex.

The analytical and structural characterization of carbohydrates or saccharides (highly abundant biological compounds) is quite a challenging problem because of the diversity of the linkage types, linkage position, and anomeric configuration [201]. Analogous to the peptide cleavage in the gas phase, the gaseous protonated/metallated oligosaccharides also undergo facile cleavages in certain specific positions upon CID. A systematic nomenclature (see Figure 19) of the different product ions produced from a precursor oligosaccharide was introduced [202] by Domon and Costello in 1988. Under low-energy collision conditions, the abundant product ions (B, C, Y, and Z) are generated by the glycosidic bond cleavages. But the high-energy collision conditions tend to favor cross ring cleavages, which produces “A” or “X” type fragments (see Figure 19) [203]. These cross-ring cleavage preferably produced by charge remote fragmentation from the sodium adduct of the precursor analyte [203]. The dominant pathway for the ion decomposition is highly dependent on the nature of the precursor ion, that is, protonated or metallated, or the cationic or anionic adduct species [203–207]. Generally for protonated oligosaccharides, the protons are mainly located on the glycosidic oxygen and thereby induce the cleavage of either C_1_–O bond or C_4_–O bond (see Figure 19) [202, 204]. The C_1_–O and C_4_–O bond cleavages give rise to the formation of B_n_/Y_m_ and C_i_/Z_j_ complementary ions, respectively, via “ion-dipole complex” mechanism involving an oxonium ion [58] (see Schemes 3 and 4).

### 8.5. CID of Oligonucleotides

**Scheme 5:**
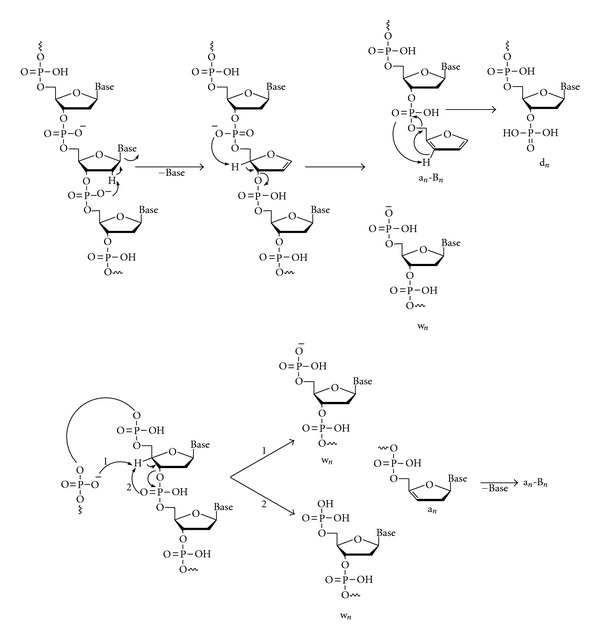
The fragmentation mechanism of the oligonucleotide in two different ways. The mechanism in the upper panel shows the necessity of a charge proximate to the cleavage site. The mechanism in the lower panel does not require the charge proximate.

**Figure 20:**
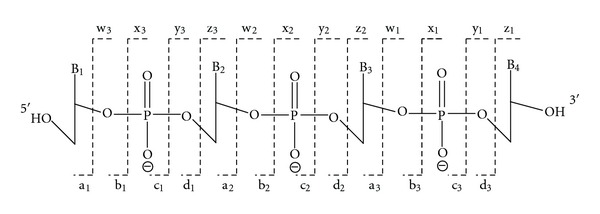
Nomenclature of the fragment ions produced by the CID of oligonucleotides.

Low energy CID is also useful for the structural elucidation of nucleic acids like DNA, RNA, and so forth, [208]. Polyanionic oligonucleotides derived from DNA/RNA can be efficiently transferred to the gas-phase by ESI. Those oligonucleotides undergo selective fragmentation in CID producing ladder-like product ion spectra similar to that produced by peptides. A systematic nomenclature was proposed by McLuckey et al. [208] to describe the product/fragment ions generated by CID of the oligonucleotide (see Figure 20). Cleavage of the four types of phosphodiester bond yields eight types of fragments for example, a_*n*_, b_*n*_, c_*n*_, and d_*n*_, which contain the 5′-OH group, and w_*n*_, x_*n*_, y_*n*_, and z_*n*_, which contain 3′-OH group. The subscript “*n*” represents the number of residues contained in the fragments and thus designates the position of cleavage. Sometimes additional loss of a purine/pyrimidine base (B*_n_*) is indicated by parenthesis with the identity of the base (if possible). For example, the fragment a_4_-B_4_ (A) indicates the cleavage of the bond between ribose carbon and the oxygen of the phosphodiester group at position 3, with the additional loss of adenosine base at the same position. ESI-MS/MS spectra of the oligonucleotides are generally dominated by a_*n*_, a_*n*_-B_*n*_, d_*n*_, and w_*n*_-ions. Other types of fragments are also possible by some complex rearrangement type reaction. Several fragmentation pathways have been proposed to explain the formation of the complimentary ions in gas phase by the precursor oligonucleotides [209–213].  Scheme 5 shows the typical mechanisms [213] of the formation of a_*n*_, a_*n*_-B_*n*_, d_*n*_, and w_*n*_-ions from the oligonucleotide anion upon CID.

### 8.6. CID of Lipids

Lipids are a broad group of naturally occurring hydrophobic organic molecules, which includes fatty acyls, glycerolipids, glycerophospholipids, sphingolipids, sterol lipids, prenol lipids, saccharolipids, and polyketides [215]. Mass spectrometry plays a key role in the structural elucidation and quantification of these molecules. It is beyond the scope here to discuss details about the CID of all classes of lipids. Here we would only discuss about the CID of two typical lipid molecules, for example, fatty acids and bile acids. Fatty acids are long hydrocarbon chain of varying length and varying degree of unsaturation, terminated by a carboxylic acid functional group. Bile acids are derived from cholesterol, and they contain a 5*β* steroid ring made up of four fused cycles bearing a side-chain attached to the C-17 carbon atom of the cycle, terminated by a carboxylic group (see Figure 22). They differ from each other by the number and position of the hydroxyl/keto groups and by the presence of unsaturation in the cycle. The bile acids can exist as free carboxylic acids or amide conjugates of the carboxylic groups with glycine (NH_2_CH_2_CO_2_H) or taurine (NH_2_CH_2_CH_2_SO_3_H).

**Scheme 6:**
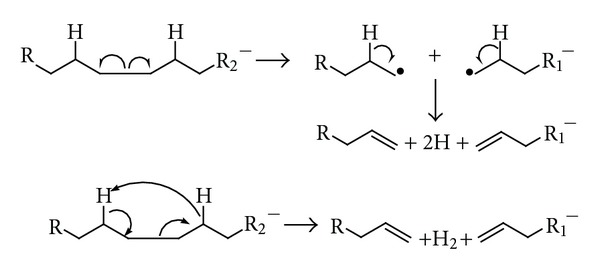
The charge remote fragmentation (CRF) mechanism for the formation of distonic ions (anions) and terminally unsaturated ions (anions).

 The fragmentation of fatty acids and bile acids is believed to occur via mechanisms that do not involve the charged group directly and thus termed as charge-remote fragmentation (CRF) mechanisms [162, 214, 216, 217]. A typical example of the CRF of a pseudomolecular anion has been shown in Scheme 6, which either involves a simple homolytic cleavage [218] or a 1,4-H_2_ elimination [216] to produce distonic radical anion or terminally unsaturated anion.

**Figure 21:**
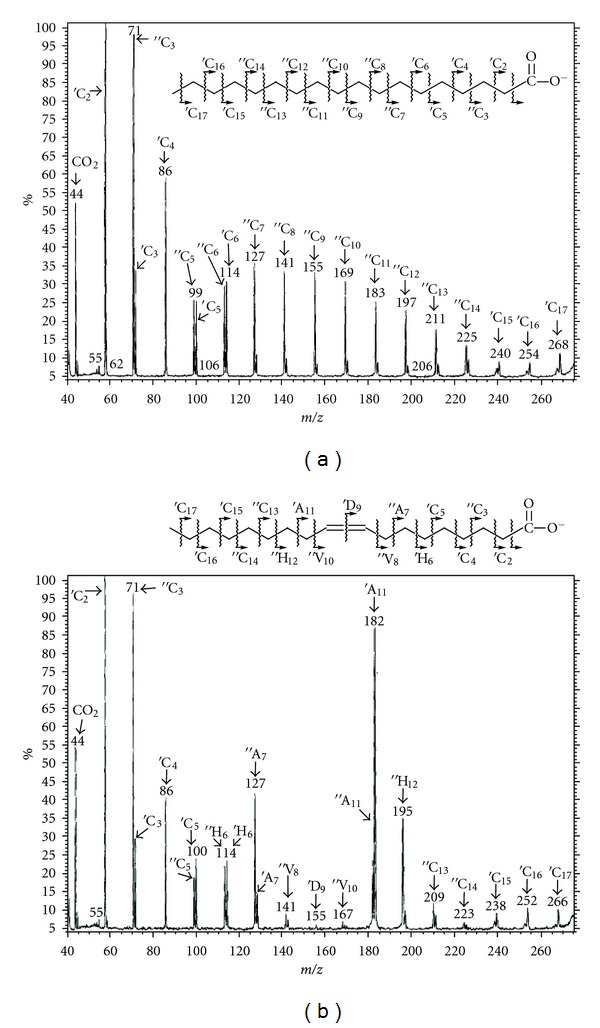
400 eV (*E*_lab_) CID spectra of stearic acid (a) and oleic acid (b) pseudomolecular anion. (Reprinted with permission from *Rapid Communication in Mass Spectrometry* [214], Copyright 1996, John Wiley and Sons).

When fatty acids are electrosprayed in negative ion mode, they produce [M−H]^−^ ions in gas phase. Upon collisional activation, the CRF of the chain in different positions occurs. According to the nature of the bond, which is broken, different symbols of the product ions are used [214]. When a regular C–C bond is broken, this is represented by C, a vinyl bond by V, an allyl bond by A, a homoallyl bond by H, and a double bond by D. A subscript to the right of the capital letter (e.g., C*_n_*) indicates the number of the carbon atoms remaining in the corresponding charged fatty acid fragment. A prime symbol to the left of the capital letter (product ion symbol) indicates that the product ion is deficient in one hydrogen atom relative to a fragment ion formed by homolytic cleavage at the same site of a hypothetical precursor molecule, and M^−•^ ([M−H]^−^ is deficient one hydrogen relative to M^−•^). Multiple hydrogen deficiencies are denoted by multiple prime symbols to the left of the letter [214].  Figure 21 shows the fragmentation characteristic of a saturated fatty acid (stearic acid) and an unsaturated fatty acid (oleic acid) under high energy collision conditions [214]. Both the fatty acids show their unique backbone fragmentation pattern.

**Figure 22:**
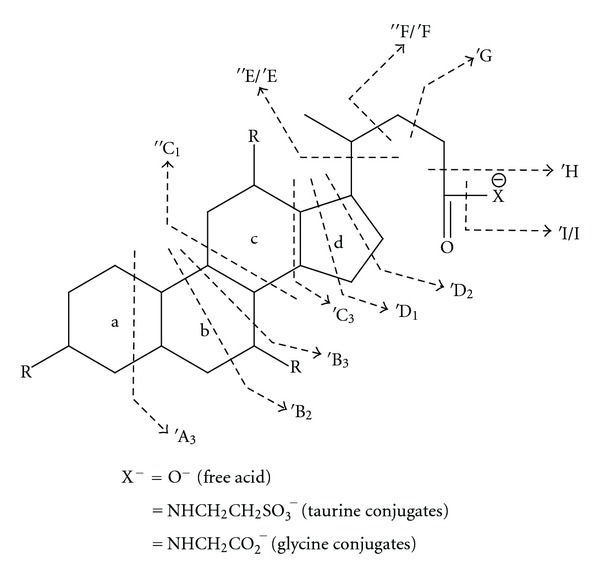
The charge remote fragmentation of bile acids (metabolites of cholesterol) with the nomenclature of different fragments.

Similarly the charge remote ring cleavage of the bile acids also occurs, resulting in ring opening, loss of neutral ethylene, and double-bond formation on the product ions. Griffiths et al. have developed a systematic nomenclature (see Figure 22) to interpret the fragment ions produced by the CID of the bile acids [217, 219]. The rings are labeled as (a), (b), (c), (d) from left to right (see Figure 22), and the corresponding capital letter is used to indicate which ring is opened and broken in the cross-ring cleavage. The subscript (1, 2, and 3) to the right side of the capital letter indicates the cross-ring cleavage site on that ring. Again the “prime” symbol to the left of the letter is used to keep track of hydrogen deficiencies as discussed above.

## 9. Application of ESI-MS

After the discovery of the ESI-MS, its application areas rapidly expanded from large macromolecules to small organic and inorganic molecules. Nowadays the ion signals in the ESI-mass spectra are providing a clear and deep perception about the analytes properties far beyond the conventional mass and structural properties of the analytes. Here we will discuss in brief about certain applications of the ESI-MS.

### 9.1. Protein Identification and Characterization

Since mass is a very specific property of a molecule, determination of the molecular weight with high precision allows to solve many problems in proteomics. Over the past twenty years, ESI-mass spectrometry has emerged as a powerful tool in the life science to determine the identity [220–222], quantity [223, 224], and structural properties [6, 225–227] of the protein molecules. Mass spectrometry is a very fast process to verify the structure and purity of the proteins and peptides [222, 228]. Classically the determination of the primary structure of the protein (i.e., the amino acid sequence) requires a chemical approach called Edman degradation method [229]. But this technique is time consuming, and a large quantity of the protein sample is also required. But after the discovery of LC-ESI-MS, the mass spectrometry is the most efficient way of sequencing a protein [107, 160, 230, 231]. In this MS method, the protein is digested by an endopeptidase (e.g., trypsin, chymotrypsin, pepsin, etc.), which cut the protein into small peptide/polypeptide fragments very specifically. Then the digest mixture is passed through the high-performance liquid chromatography where the collection of peptide fragments is separated. At the end of the column the individual peptide fragment is electrosprayed in vacuum, where they are trapped and allowed to undergo collision activation to produce MS/MS spectra as discussed above [160, 231]. The resulting MS/MS spectra are nothing but the mass fingerprints of the ladder-like product ions of the peptide of a particular sequence. The MS/MS spectra of the peptides are usually assigned with the help of various bioinformatic tools that implement sequencing algorithms based on peptide fragmentation chemistry as discussed before [160, 231, 232]. Thus the peptide mass fingerprinting (PMF) helps to identify the amino acid sequence of the whole proteins. The process is repeated using different digestion enzymes, and the overlaps of the resulting sequences are used to construct a sequence of the protein. When the identification of the proteins is solely based on the sequence data obtained from the tandem mass analysis, it is called “de novo sequencing” [232], and the corresponding procedure (as discussed above) of the protein analysis is called “bottom-up” approach [233, 234].

 Sometimes intact charged protein generated by ESI is introduced into the mass analyzer and are subjected to gas-phase fragmentation, and this type of approach is referred to as the “top-down” strategy of protein analysis [235].

 Although the ESI-MS technique is not sensitive to probe the local conformation of the polypeptide chain, that is, the secondary structure of the protein, the technique can successfully interpret the three-dimensional conformation of the proteins [76]. As discussed already that the charge state distribution actually represents the three dimensional folding or the tertiary structure of the proteins [76]. This property makes ESI-MS an excellent method, complimentary to CD (circular dichroism), to characterize the protein conformational change. Hence an increasing interest based on ESI-MS is continuously developing to study the protein folding process.

 ESI-MS can also successfully detect the posttranslational modifications (PTMs) [236, 237] and the mutation [238] of the proteins as those processes lead to the change of protein masses. Not only does the total mass change, but also the position or point of the PTM or mutation can be determined by tandem mass spectrometric experiments [222, 239]. Recently, using tandem mass spectrometric technique, we have identified the reactive lysine residues of cytochrome c [240]. We found that the lysine residues, which are in the turn or loop region of the protein, are more reactive to succinylation compared to those which are in the helical region. It has been proposed that the lower reactivity of the lysine residues present in the helical regions might be due to the higher rigidity of the helical region than that of the turn or loop region [240]. In another study [241], using tandem mass spectrometry we have determined the molecular basis of the PTM involving covalent attachment of the heme with a glutamic acid of the protein matrix in the Cytochrome P450 enzyme.

 Disulphide bonds (a kind of PTM) play important roles in the structure and biological activity of the cystinyl proteins. So the determination of the disulphide bond linkage between two adjacent or closely spaced cysteine residues is an integral part of the structural characterization of the proteins. Several mass spectrometry-based strategies have been developed to map the disulphide bond linkages in the proteins [242–247]. Most of the time, the protein of interest is cleaved enzymatically in its nonreduced states, and then the resulting disulphide-linked peptides are separated, identified, and characterized by LC-MS technique. Then the data is compared with the similar experiments performed on the disulphide-reduced protein of the interest [243]. This comparison study can locate the disulphide linkages in the folded proteins. Sometimes chemical cleavage at disulphide residues followed by chemical derivatization is also performed for the mass spectrometric study to identify the disulphide linkages in the proteins [247].

### 9.2. Studying Noncovalent Interaction

Since ESI is a sufficiently soft ionization technique, the noncovalent complexes of the analytes formed in solution can representatively, transferred to the gas phase when appropriate instrumental conditions are used [225, 248–250]. Mainly a collection of weak interactions such as Van der Waals forces, hydrophobic forces, and hydrogen bonding or salt bridges (electrostatic interactions) are responsible for the analyte association in solution. When the analyte is transformed in the gas-phase via ESI, probably most of those interactions are retained, and some of those interactions become more prominent in the gas phase compared to solution and thus provide the structural integrity in the gas phase [251–253]. Generally the propensities of the ionic interactions become more in the gas phase compared to solution [251–253]. For example, a recent ESI-MS study of the protonated and deprotonated gaseous ions of a single chain antibody-trisaccharide complex has showen several specific intermolecular H-bonds in the gas phase [254]. Likewise a recent report, shown that, in the presence of K^+^, Rb^+^, and Cs^+^, uracil, thymine, and their homologues form self-assembled quintet structures that are stabilized by hydrogen bonding and ion-dipole interactions in the gas phase [255]. ESI-MS has allowed the observation of a large number of biomolecular noncovalent complexes such as protein-protein [256, 257], protein-metal ion [258], protein-drug [259], and protein-nucleic acid [260] complexes. Since proteins provide a large number of functional groups for the noncovalent interaction with the partner molecule(s), the resultant noncovalent forces are large enough to retain the association during their transfer from condensed to the gas phase. But, in small molecules for example, in small peptides, this number of noncovalently interacting atoms with proper orientation for the intermolecular interaction is less and it is sometimes very difficult to probe their association in solution by ESI technique. Recently we have shown the formation of noncovalent dimers of the lysine containing basic peptides by ESI-MS [253], which demonstrated that the intermolecular electrostatic/H-bonding network is mainly responsible for the dimer formation of those small peptides in the gas phase. ESI-mass spectrometry also evolved as a valuable tool for the determination of the association/dissociation constants for several protein-ligand complexes [261]. Although the gas-phase studies on the proterin-ligand complexes have been primarily focused on the complexes, which are stabilized by the ionic interaction or H-bonding, the nonpolar intermolecular interaction between protein (bovine *β*-lactoglobulin) and ligand (fatty acids) in vacuum has also been reported lately [262]. The corresponding interaction strength of the nonpolar protein-ligand complexes has also been quantified.

It is very difficult to ionize the hydrophobic proteins by ESI because of their inherent insolubility in the buffers compatible with electrospray. For this reason the ESI-mass spectrometry has not been applied to intact membrane protein complexes. But recently Barrera et al. have been successful to transfer some hydrophobic membrane protein complexes in vacuum via ESI by encapsulation in a solution phase detergent micelle [263].

The ESI-MS has also been used to study the complexes between polyether (e.g., crown ethers) and protonated peptides [264, 265]. The protonated amine functional groups make hydrogen bonds with the oxygen atoms of the crown ethers in those complexes. The energy-variable collision-induced dissociation was carried out to analyze the strengths of noncovalent interactions of protonated peptide/polyether complexes [264, 265].

### 9.3. In Clinical Laboratory

Since ESI-MS is a sensitive, robust, and reliable tool for studying the femtomole samples in microlitre volumes, it has become an increasingly important technique in the clinical laboratory for structural study or quantitative measurement of metabolites in a complex biological sample [266]. For example, HPLC/ESI-MS is useful to a great extent than other conventional techniques in screening for inborn errors of amino acid [267, 268], fatty acid [269], purine [270], pyrimidine [270] metabolism, and diagnosis of galactosaemia [271] and peroxisomal [272, 273] disorders. Because of the preservation of the noncovalent interaction in gas phase, ESI-MS has nurtured a new and improved approach (versus electrophoresis) for identification and quantification of haemoglobin variants [274]. With the understanding of glycohaemoglobin structure, an IFCC reference method for glycohaemoglobin assay has been established using ESI-MS [275]. It also represents a promising strategy for the standardisation of HbA1c in diabetic monitoring [276]. With its other applications such as in therapeutic drug monitoring and identification of biomarkers [277, 278], ESI-MS will continue to exert a more important influence in the clinical laboratory in near future.

### 9.4. Probing Molecular Dynamics: H/D-Exchange Experiments

The application of the ESI-MS is not only restricted to the structural characterization, but it has also been extended recently to the study of the molecular dynamics. For this type of application, the analyte ions produced by the ESI-ion source are trapped and subjected to the ion-molecule reaction (H/D exchange) with some gaseous deuterated molecules possessing exchangeable deuterium (e.g., CH_3_OD, ND_3_, etc.) for well-defined reaction intervals in the collision cell [279]. As a result the H/D-exchange between analyte and the deuterated molecules occurs in the gas phase and thus provides some critical information regarding the molecular motion in the noncovalent complexes. For example, the H/D exchange reactions have been applied to uncover the tumbling motion of ammonium guests bound inside the cavity of resorcinarene hosts [280]. Very recently a highly dynamic motion of the crown ethers along the oligolysine peptide chains has been probed by H/D-exchange experiments in the gas phase [281]. The authors suggested this phenomenon as the wire dance on the molecular level. It has been observed that the crown ethers (guest molecules) can move directly between different binding sites of the oligolysine (a multitopic host) without intermediate dissociation. Furthermore, the exchange experiments unambiguously revealed the zwitterionic structure of the crown ether/oligolysine complexes, highlighting the success of the gas-phase experiments for investigating noncovalent interactions [281].

 The gas-phase folding and unfolding of the protein (protein dynamics) can also be monitored by the H/D-exchange experiments [282, 283]. Valuable information regarding the protein conformation in vacuum has also come out by the gas-phase isotope exchange experiments [138, 284, 285]. Similarly several ion-molecule reactions in gas-phase have been used for the covalent modification of the gaseous analyte [286].

### 9.5. Monitoring Chemical Reactions and Studying Reactive Intermediates

The applications of ESI-MS have also been explored in synthetic organic and organometallic chemistry to study the reactive intermediates and the mechanisms [287–292]. For example, catalytic intermediate of the Suzuki coupling reaction [289] and the Heck reaction mechanism [291] has been studied by ESI-MS. Raney nickel-catalyzed coupling reaction of 2-bromo-6-methylpyridine was studied, and a reactive dimer of the intermediate Ni[II] complex of 6,6′-dimethyl-2,2′-bipyridine was detected [293]. Desorption electrospray ionization mass spectrometry (DESI-MS) has been used for monitoring solid-state organic reaction in ambient air, specifically the Bayer-Villiger type reactions involving the oxidation of ketones by m-chloroperbenzoic acid in solid state [294]. The 1-adamantyl radicals have been identified as a reactive intermediate in several organic syntheses. Recently a tert-adamantyl peroxyl radical has been trapped in gas phase using ESI-MS, and its unusual structure and reactivity has been investigated [295]. Many other types of the reaction mechanisms investigated by the ESI-MS technique have recently been reviewed by Eberlin [296].

### 9.6. Chemical Imaging

Desorption electrospray ionization mass spectrometry (DESI-MS) is relatively a new ambient surface analysis method. The development of imaging mass spectrometry by DESI has been described recently [297–299], and its application to high-throughput biological tissue imaging was also demonstrated [298]. In this technique the spatial distribution (on the tissue surface) as well as structural identification of the molecule of interest can be accomplished successfully [298]. Chemical imaging by DESI has been applied to label-free detection of drugs and metabolites in tissue [298]. DESI-MS imaging is advantage in a number of ways over the conventional whole-body autoradiography approach: (1) no radioactive label is required and (2) it allows simultaneous detection of the parent drug compound and metabolites in tissue [300]. DESI also holds the advantages of speed and specificity inherent in the mass spectrometric experiment. Chemical imaging by DESI-MS is still, however, in its early childhood. The ongoing research activities in this area address the questions concerning sensitivity, compound-specific ionization yields, tissue-specific ion suppression, and effects of solvent composition on ionization yields. An insight on these issues would immensely widen the horizon of applications of this technique in assessing more complex and quantitative information on the samples that would have important medical and pharmaceutical implications.

## 10. Future Prospects and Outlook

The development of electrospray ionization mass spectrometry has enabled us to solve a wide range of biochemical and mechanistic problems as discussed above. Perhaps it is one of the instruments that has continuously evolved over the last three decades both in application and modification of instrument design. For example, the transition of source design from microspray to nanospray has improved the sensitivity. Introduction of high-resolution mass analyzer (FT-ICR) amended the accuracy and redefined the applicability.

 Yet in several aspects, the understanding of the ESI-MS remains vague to date. One of the major issues is the exact mechanism of ion formation. How is the gas-phase ion formation guided mechanistically? Is the perception of CRM and IEM enough to interpret the ion formation? It is still unclear about the analyte behavior (structure and dynamics) inside the charged droplets produced by ES process. If the proteins change their structure inside the charged droplet, then the charge state distribution (CSD) should reflect the instantaneous conformation of the protein inside the charged droplet not in the original solution. Again, as discussed in the present review, the CSD is not influenced by a specific parameter but governed by multiple parameters. So the precise understanding of the cumulative effect of those different parameters on the protein CSD would no doubt help to earn more quantitative insights about structural and chemical behavior of the proteins.

So far a little attention was invoked on the detector characterization and improvement compared to that of the ion source and mass analyzer. So, the characterization and improvement of the detector would likely be the next step in the development of mass spectrometry which would enable us to give the answer whether the ion signal intensity is governed by the molecular conformation of the analyte or not.

 The application of the ESI-MS got a noteworthy dimension in biochemistry laboratories after its success in protein sequencing. The sequencing is based on the idea and information of gas-phase ion fragmentation chemistry of the peptides in the tandem mass spectrometry. But the tandem mass spectra of the peptides are mostly dominated by anomalous fragment ions than the “sequence informative ions.” So the characterization of those anomalous fragments and accordingly the modification of sequencing algorithms would facilitate the sequencing of proteins/peptides more precisely and rapidly using mass-spectrometric technique.

## Figures and Tables

**Table 1:** Typical redox reactions of the solvents expected to occur in the electrospray emitter and their corresponding standard potentials.

Solvent systems	Positive-ion mode		Negative-ion mode	
	Oxidation reactions	*E * ^0^ (V)	Reduction reactions	*E * ^0^ (V)

Water	2H_2_O = O_2_ + 4H^+^+ 2e^−^	1.23	2H_2_O + O_2_ + 4e^−^ = 4OH^−^	0.40
	2H_2_O = H_2_O_2_ + 2H^+^+ 2e^−^	1.77	H_2_O + O_2_^−^ + e^−^ = HO_2_^−^ + OH^−^	0.20
	H_2_O = HO* + H^+^+ e^−^	2.72	H_2_O + HO_2_^−^ + e^−^ = HO* + 2OH^−^	0.18
			2H_2_O + 2e^−^ = H_2_ + 2OH^−^	0.07
			2H_2_O + O_2_ + 2e^−^ = H_2_O_2_ + 2OH^−^	−0.13
			H_2_O + O_2_ + 2e^−^ = HO_2_^−^ + OH^−^	−0.83

Methanol	CH_3_OH = HCHO + 2H^+^ + 2e^−^	0.23	CH_3_OH + H_2_O + 2e^−^ = CH_4_ + 2OH^−^	−0.25
	CH_3_OH + H_2_O = HCOOH + 4H^+^ + 4e^−^	0.10	CH_3_OH + 2H^+^ + 2e^−^ = CH_4_ + H_2_O	0.58
	HCOOH = CO_2_ + 2H^+^ + 2e^−^	−0.20		
